# Single-Molecule Insights Into the Dynamics of Replicative Helicases

**DOI:** 10.3389/fmolb.2021.741718

**Published:** 2021-08-26

**Authors:** Richard R. Spinks, Lisanne M. Spenkelink, Nicholas E. Dixon, Antoine M. van Oijen

**Affiliations:** ^1^ Molecular Horizons and School of Chemistry and Molecular Bioscience, University of Wollongong, Wollongong, NSW, Australia; ^2^ Illawarra Health and Medical Research Institute, Wollongong, NSW, Australia

**Keywords:** helicases, dynamics, replisome, DNA replication, single-molecule, fluorescence, multi-protein complexes

## Abstract

Helicases are molecular motors that translocate along single-stranded DNA and unwind duplex DNA. They rely on the consumption of chemical energy from nucleotide hydrolysis to drive their translocation. Specialized helicases play a critically important role in DNA replication by unwinding DNA at the front of the replication fork. The replicative helicases of the model systems bacteriophages T4 and T7, *Escherichia coli* and *Saccharomyces cerevisiae* have been extensively studied and characterized using biochemical methods. While powerful, their averaging over ensembles of molecules and reactions makes it challenging to uncover information related to intermediate states in the unwinding process and the dynamic helicase interactions within the replisome. Here, we describe single-molecule methods that have been developed in the last few decades and discuss the new details that these methods have revealed about replicative helicases. Applying methods such as FRET and optical and magnetic tweezers to individual helicases have made it possible to access the mechanistic aspects of unwinding. It is from these methods that we understand that the replicative helicases studied so far actively translocate and then passively unwind DNA, and that these hexameric enzymes must efficiently coordinate the stepping action of their subunits to achieve unwinding, where the size of each step is prone to variation. Single-molecule fluorescence microscopy methods have made it possible to visualize replicative helicases acting at replication forks and quantify their dynamics using multi-color colocalization, FRAP and FLIP. These fluorescence methods have made it possible to visualize helicases in replication initiation and dissect this intricate protein-assembly process. In a similar manner, single-molecule visualization of fluorescent replicative helicases acting in replication identified that, in contrast to the replicative polymerases, the helicase does not exchange. Instead, the replicative helicase acts as the stable component that serves to anchor the other replication factors to the replisome.

## Introduction

The chemical directionality of DNA poses a unique challenge when it comes to replicating the chromosomes of an organism. The replisome, the protein complex responsible for DNA replication, must closely coordinate DNA synthesis occurring in opposite directions on each of the two strands, with DNA unwinding occurring in the direction of fork progression. In all organisms from viruses to humans, DNA replication is an essential task, but the replisome architecture has diverged significantly (Yao and O’Donnell, 2016a). Even though all replisomes contain a replicative helicase, the bacterial and eukaryotic equivalents are not homologs, suggesting that the enzyme responsible for DNA unwinding evolved twice, independently (Leipe et al., 1999). Yet these helicases have demonstrated functionally similar properties and are expected to fulfil similar roles within the replisome (Yao and O’Donnell, 2016b; Brosh and Matson, 2020). This type of convergent evolution implicates the replicative helicase as one of the most critically important components of the replisome.

Since the initial identification of replicative helicases, research efforts of the last 35 years have sought to characterize the functional properties of these enzymes. Significant headway has been made to determine helicase structure and assembly, as well as directionality, chemical-energy turnover (nucleotide hydrolysis), and nucleic-acid specificity (summarized in Perera et al., 2019). While these studies have resulted in highly refined models of unwinding, an exact mechanistic understanding of the process remains incomplete. Details such as active conformational state, coordination of their subunits, and the size of each step are poorly understood due to the lack of experimental accessibility. These gaps in our knowledge led researchers to develop high resolution, single-molecule tools capable of manipulating and observing helicase activity and thus accessing intimate mechanical details of these proteins. In parallel, development of novel fluorescence visualization methods has likewise made it possible to observe DNA replication at the single-molecule level both *in vitro* and *in vivo*. Focusing such methods on replicative helicases has provided a unique window to observe intra- and inter-molecular variation in helicase activity within the context of the replisome.

In this review, we compile the latest single-molecule insights into the function of replicative helicases and discuss the current thinking on unwinding mechanisms as well as their roles in the replisome. We focus on the replicative helicases of the model viral species, bacteriophages T4 and T7, the model bacterial species *Escherichia coli*, and the model eukaryotic species, *Saccharomyces cerevisiae*. We compare universal helicase traits across this diverse set of organisms and identify similarities and differences.

## The Single-Molecule Toolbox to Examine Helicases

In the last two decades, a number of biophysical methods have been developed that permit the observation and manipulation of single molecules. These innovations were initially concentrated on specific mechanistic questions, but the popularity of these methods has since given us a fundamentally new understanding of molecular biology (van Oijen and Dixon, 2015). Such methods are not the focus of this review, but their importance will be briefly discussed here.

Conventional ensemble techniques have proven extremely useful; however, single-molecule approaches offer advantages in several key aspects. Being a direct measure of a molecule’s properties, these methods circumvent the effects of ensemble averaging, and thus can detect stochastic variations in molecular activity as well as transient intermediate states. Combining the measurements on many single molecules shows the complete distribution of a molecular population as well as rarer sub-populations that are otherwise difficult to capture.

**FIGURE 1:**
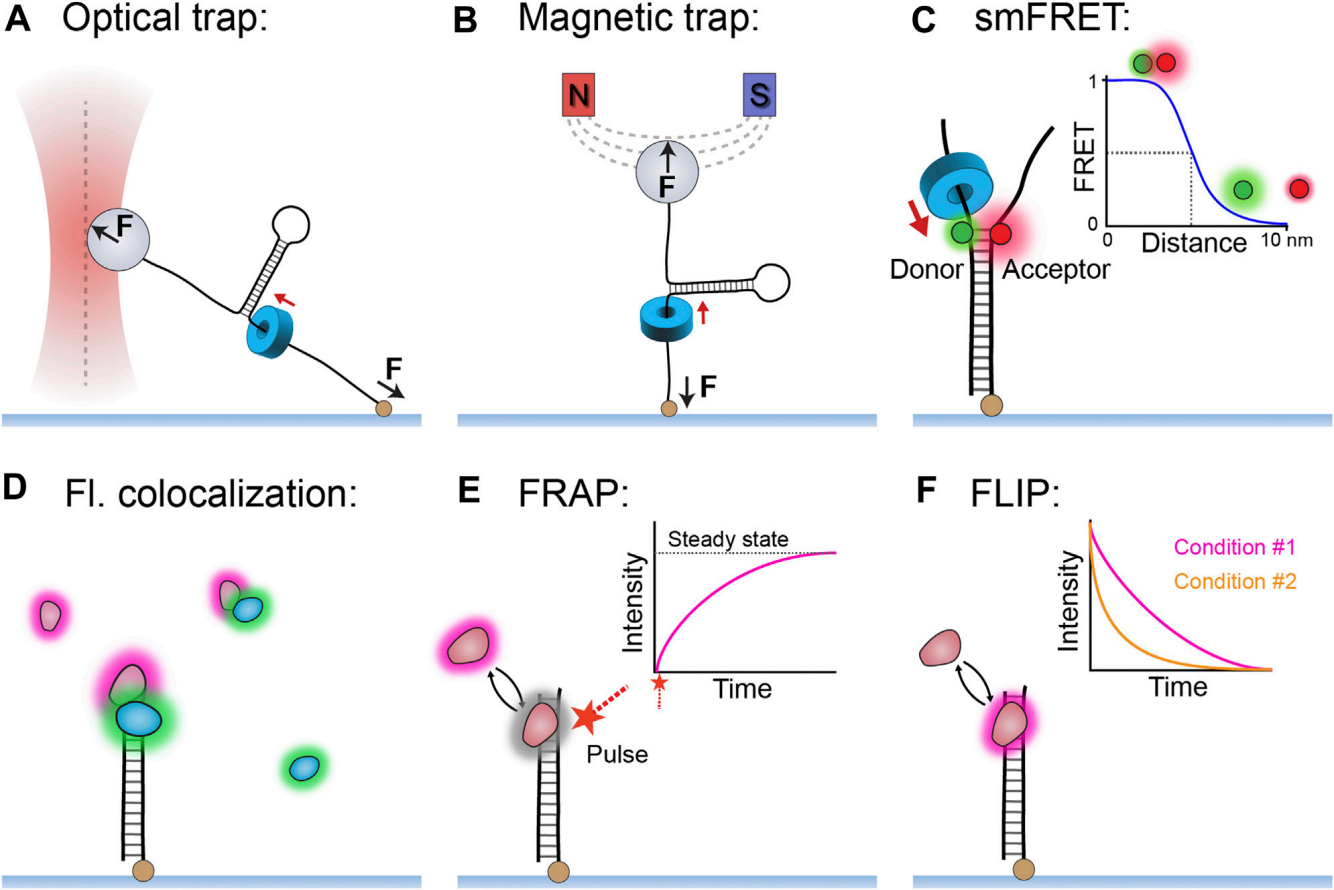
**Single-molecule methods used to study replicative helicases. (A)** An optical trap uses a laser to apply a pulling force (0.5–100 pN) to a dielectric bead, which can in turn apply force to a DNA template and measure changes in DNA length during helicase unwinding. **(B)** A magnetic trap uses a magnetic field to apply force (10–100 pN) and torque to a paramagnetic bead and thus measure DNA length changes during helicase unwinding. **(C)** Single-molecule FRET can measure helicase unwinding through the changes in proximity of the donor and acceptor fluorophores positioned within the DNA template. (Inset) The relationship between FRET efficiency and distance between the fluorophores. **(D)** The simple technique of multi-color colocalization is effective at detecting helicase interactions in complex reactions. **(E)** Single-molecule fluorescence recovery after photobleaching (FRAP) method can be used to quantify protein exchange in the form of recovered fluorescence following a deliberate bleaching event. **(F)** Single-molecule fluorescence loss in photobleaching (FLIP) method can also detect protein exchange when comparing different fluorescence lifetimes between conditions.

Single-molecule techniques can be broadly split into two classes: those based on applications and measurements of force and those based on detection of fluorescence. For the single-molecule study of helicases, the most used force techniques are optical and magnetic traps (reviewed in detail by Miller et al., 2018). An optical trap consists of a tightly focused laser that creates an electromagnetic gradient to pull a dielectric bead to the focal point of the gradient (Figure 1A). Similarly, a magnetic trap generates a magnetic-field gradient to apply force and torque to a paramagnetic bead (Figure 1B). In a typical helicase experiment, a trap pulls on a bead (low forces of ∼5–10 pN) attached to an immobilized DNA template. Subsequently, single events of helicase unwinding can be measured through DNA length changes. Furthermore, both optical and magnetic traps can be used to manipulate the DNA with high forces (>20 pN) and observe how the helicase acts on DNA that is destabilized at the site of unwinding.

There is a diverse range of single-molecule fluorescence methods, of which several have been particularly effective for studying helicase function (reviewed in detail by Miller et al., 2018). The method of fluorescence resonance energy transfer (FRET) — where energy transfer correlates with fluorophore proximity—has provided a high-resolution readout of DNA unwinding or other conformational changes (Figure 1C). Also of note are the multi-color colocalization methods that, although conceptually simple, are highly effective at reporting on the interaction network of replicative helicases even in complex reactions (Figure 1D). The single-molecule methods of fluorescence recovery after photobleaching (FRAP; Figure 1E) and fluorescence loss in photobleaching (FLIP; Figure 1F) have also been useful in replicative helicase studies. FRAP and FLIP respectively measure the appearance and disappearance of fluorescence as a result of molecular exchange and thus make it possible to quantify this process for helicases both *in vitro* and *in vivo*. FRAP is generally the more versatile of the two methods, as it measures a very obvious reappearance of fluorescence after deliberate bleaching as an indicator of exchange.

## Model Replisomes and Their Helicases

### Phage T4 gp41 and Phage T7 gp4 Helicases

**FIGURE 2:**
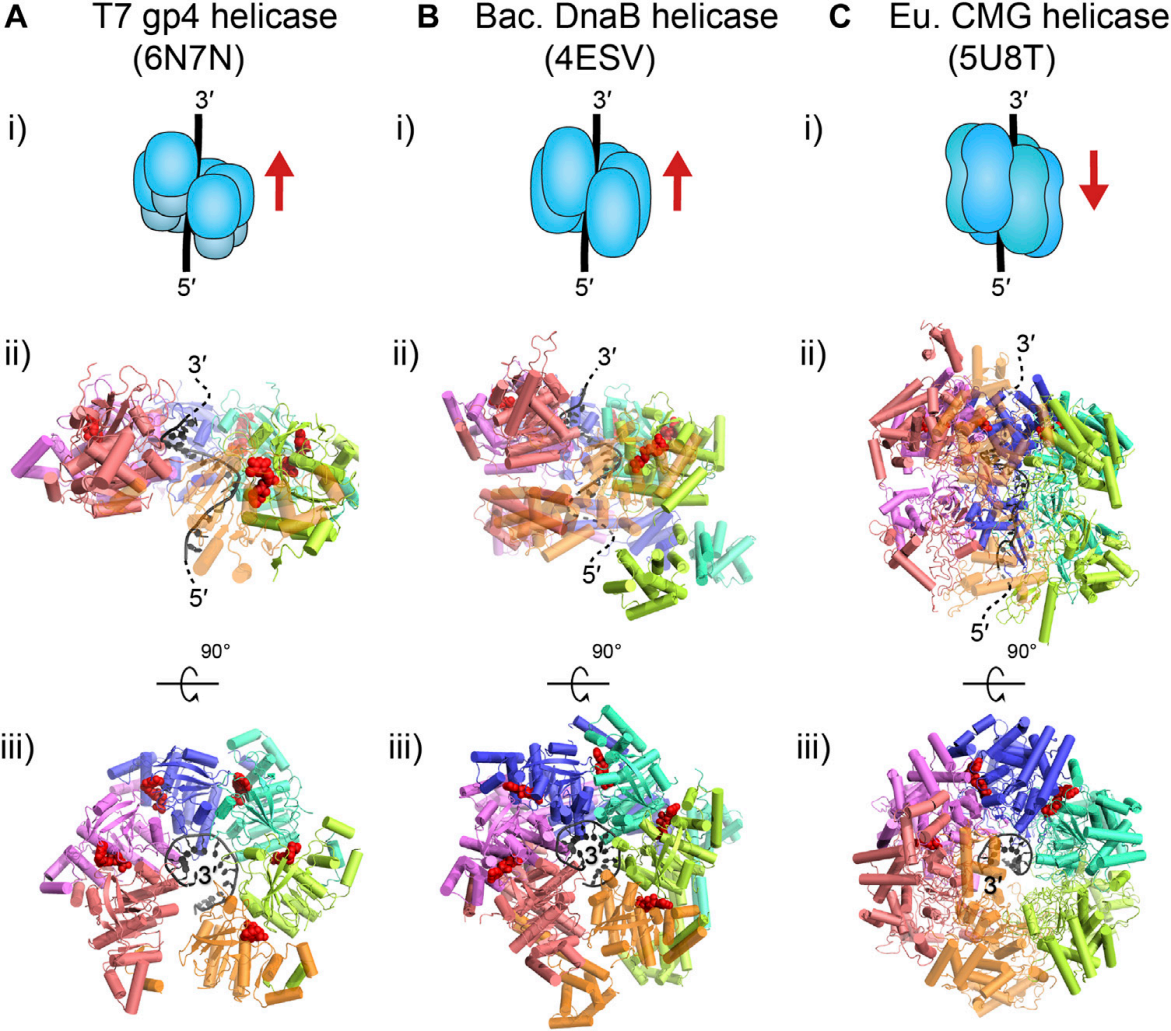
**Translocation states of the replicative helicases of interest.** T7 gp4 **(A)** and *E. coli* DnaB **(B)** are homo-hexamers that move along ssDNA in the 5′–3′ direction **(i)**. Both of these prokaryotic, homologous helicases are thought to translocate in a spiral staircase conformation, where each subunit contacts 2 nt and moves sequentially along DNA **(ii)**; gp4: PDB ID: 6N7N (note: the gp4 N-terminal primase domain in the structure has been omitted); DnaB: PDB ID: 4ESV (note: the last orange subunit is semi-transparent). The top view of these structures **(iii)** shows that each subunit binds a nucleoside triphosphate molecule (red) at the subunit interface, except at the opening at the first (pink) and last (orange) subunit. Note, structures of the T4 gp41 helicase are currently not available but are expected to be similar to gp4 (Mueser et al., 2010). The eukaryotic CMG replicative helicase **(C)** is a hetero-hexamer that moves along ssDNA in the 3′–5′ direction **(i)**. The MCM2–7 hexamer of CMG is expected to translocate in a bridged spiral conformation with the final subunits closing the gap **(ii)**; CMG: PDB ID: 5U8T (note: Cdc45 and GINS are omitted here, and the last orange subunit is semi-transparent). The top view of CMG **(iii)** shows only two subunits binding nucleotides (red), even though all subunits have binding pockets.

The model bacteriophages T4 and T7 provide the simplest forms of the replisome. In these species, the replicative helicases, gp4 in T7 and gp41 in T4, are superfamily (SF) four helicases, which are characterized as homo-hexameric rings that employ a RecA-like motor domain to power translocation along single-stranded (ss) DNA in the 5′–3′ direction and concomitantly unwind duplex DNA by exclusion of the other strand. For T7, the gp4 translocation state is observed as a spiral staircase offset around single-stranded DNA (ssDNA) that structurally resembles one strand of A-form DNA, where each subunit contacts the backbone with a footprint of two nucleotides (nt) and with the nucleoside triphosphatase (NTPase) site holding an NTP at the interface between subunits (Figure 2A) (Gao et al., 2019). For T4, there are currently no structures available of gp41; however, sequence-based predictions of the structural motifs suggest a hexameric arrangement very close to that of gp4 helicase (Mueser et al., 2010).

**FIGURE 3:**
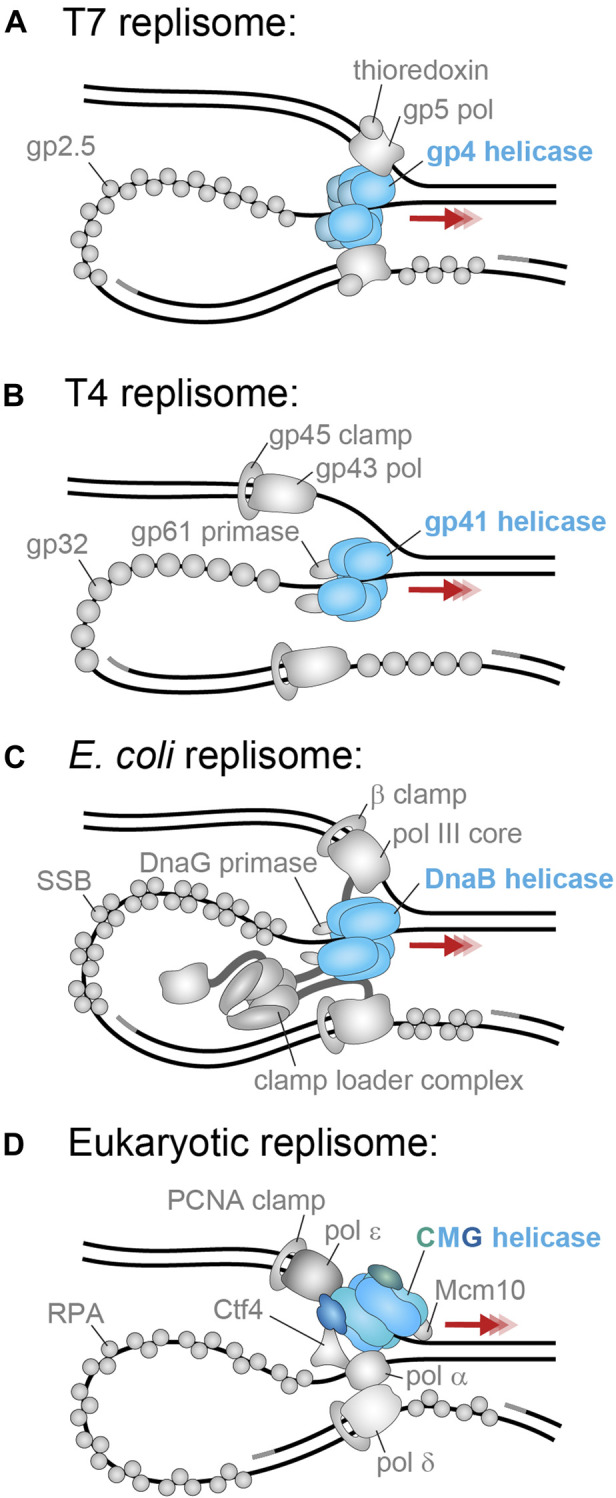
**The best-studied replisomes and their helicases. (A)** The T7 phage replicative helicase, gp4 (blue), translocates in the 5′–3′ direction on the lagging strand and contacts the leading and lagging strand polymerases (Scherr et al., 2018). **(B)** The T4 phage replicative helicase, gp41 (blue), translocates in the 5′–3′ direction on the lagging strand and contacts only the gp61 primase (Benkovic and Spiering, 2017). **(C)** The *E. coli* replicative helicase, DnaB (blue), translocates in the 5′–3′ direction on the lagging strand and contacts the DnaG primase and the τ subunit of the leading- and lagging-strand arms of the clamp loader complex (Lewis et al., 2016). **(D)** The *S. cerevisiae* replicative helicase, CMG (Cdc45 in green; MCM2–7 in blue, GINS in dark blue), translocates in the 3′–5′ direction on the leading strand and contacts the leading strand polymerase Pol ε, as well as the primase Pol α, firing factor Mcm10, organizing factor Ctf1, and MTC accessory factor (omitted) (Lewis et al., 2020).

These replicative helicases have different ways of interacting with the primase enzyme that promotes the synthesis of short RNA primers on the lagging strand. In the T7 replisome (Figure 3A), the primase is fused to the helicase and forms the N-terminal domain tier of gp4, but in the T4 replisome (Figure 3B), the primase is a separate protein (gp59) that binds to the gp41 helicase. Similarly, in each replisome the replicative helicase interacts with both the leading- and lagging-strand polymerases. The T7 gp4 helicase maintains a direct, physical connection to the gp5 polymerase. In T4, the gp43 polymerase is known to interact with the gp41 helicase but only transiently with lagging-strand replication likely occurring away from the fork. For a detailed review of T4 replication, see Benkovic and Spiering (2017) and for T7 replication see Kulczyk and Richardson (2016).

### *E. coli* DnaB Helicase

The *E. coli* replicative helicase, DnaB, is homologous to its bacteriophage counterparts, as it is also a homo-hexameric RecA-like helicase of the SF4 group. DnaB also uses its ATPase ability to translocate in the 5′–3′ direction on ssDNA and unwind double-stranded (ds) DNA. Similar to T7 gp4, the DnaB translocation state is also observed as a spiral staircase with each subunit holding a NTP at the subunit interface and in contact with 2 nt of the A-form-like DNA backbone (Figure 2B) (Itsathitphaisarn et al., 2012). It is important to note that this information is based on DnaB structures not from Gram-negative *E. coli*, but from the thermophilic Gram-positive species, *Geobacillus stearothermophilus*. However, the tertiary and quaternary structure is very similar to another, non-translocation state structure of DnaB in complex with DnaC from *E. coli* (Arias-Palomo et al., 2019).

Within the *E. coli* replisome (Figure 3C), DnaB interacts with 1–3 DnaG primase molecules via three binding sites on pairs of the helicase N-terminal domains. It is unclear if this interaction is constant or only transient during moments of primer synthesis and hand-off. Additionally, the DnaB helicase maintains a physical coupling to the polymerase III holoenzyme (leading and lagging Pol III cores, their associated β clamps, and the clamp loader complex) via the τ subunit of the clamp loader complex. It is not known where the τ subunit binds on the DnaB helicase, but it is expected that the strength of this interaction varies during replication as a means to direct primer hand-off and polymerase exchange (Monachino et al., 2020). For a detailed review of *E. coli* DNA replication, see Lewis et al. (2016).

### *S. cerevisiae* CMG Helicase

The replicative helicase in the model eukaryote *S. cerevisiae*, is CMG, which consists of three parts: the Cdc45 activating factor, the MCM2–7 hetero-hexameric ring, and the hetero-tetrameric GINS. CMG is a SF6 helicase with as the main component, the MCM2–7 ring, which contains a AAA+ motor domain to power translocation along ssDNA and unwinding of double-stranded DNA (dsDNA). From structures of CMG bound to ssDNA, we see MCM2–7 form a partial spiral around A-form-like DNA, with the final two subunits closing the spiral (Figure 2C) (Georgescu et al., 2017). The C-terminal motor domain sits behind the N-terminal domain and pushes it in the 3′–5′ direction on ssDNA.

Due to this reversed directionality of CMG relative to the bacterial helicases, it translocates on the leading strand and excludes the lagging strand (Figure 3D). Therefore, the leading-strand polymerase Pol ε synthesizes DNA directly behind CMG and is expected to contact both GINS and the rear of MCM2–7. The lagging-strand polymerase Pol δ does not directly contact CMG. It is expected to either act behind the replisome or interact with CMG indirectly via the primase Pol α, or the Ctf4 organizing protein. The MTC complex also interacts with CMG to connect the helicase to multiple other replisome components. Furthermore, CMG has an essential yet ambiguous interaction with the Mcm10 firing factor which seems to enhance translocation and unwinding. A detailed review of the eukaryotic replisome can be found in Lewis and Costa (2020).

## Novel Insights Into Helicase Mechanisms

### Active Versus Passive Helicases

Ensemble biochemical studies have characterized replicative helicases and put forward the idea that these enzymes are universally poor motors when removed from their respective replisomes (Delagoutte and von Hippel, 2002; Delagoutte and von Hippek, 2003). The ensemble-averaging inherent in these methods, however, makes it challenging to uncover the precise molecular mechanisms underlying helicase activity. The principles that govern the rate of unwinding and the processivity of replicative helicases have not been fully elucidated, nor is it clear how the poor unwinding activity of isolated helicases is reconciled with the highly efficient unwinding occurring within replisomes. Single-molecule micro-manipulation techniques are very useful in this respect, as they can detect single events of unwinding with high sensitivity and offer the ability to apply force to the DNA to see how such mechanical manipulation influences enzymatic activity.

Several single-molecule studies were able to quantify individual unwinding events on tethered DNA templates using optical traps for the gp4 helicase (Johnson et al., 2007; Sun et al., 2011; Sun et al., 2015; Sun et al., 2018), and magnetic traps for the gp41 helicase (Lionnet et al., 2007; Manosas et al., 2010; Ribeck and Saleh, 2013), DnaB helicase (Ribeck et al., 2010) and CMG helicase (Burnham et al., 2019). All of these studies share an experimental design where a DNA template is tethered at one end to a glass slide and the other end to a bead that is manipulated by an optical or magnetic trap (Figures 1A,B; see *The Single-Molecule Toolbox to Examine Helicases*).

**FIGURE 4:**
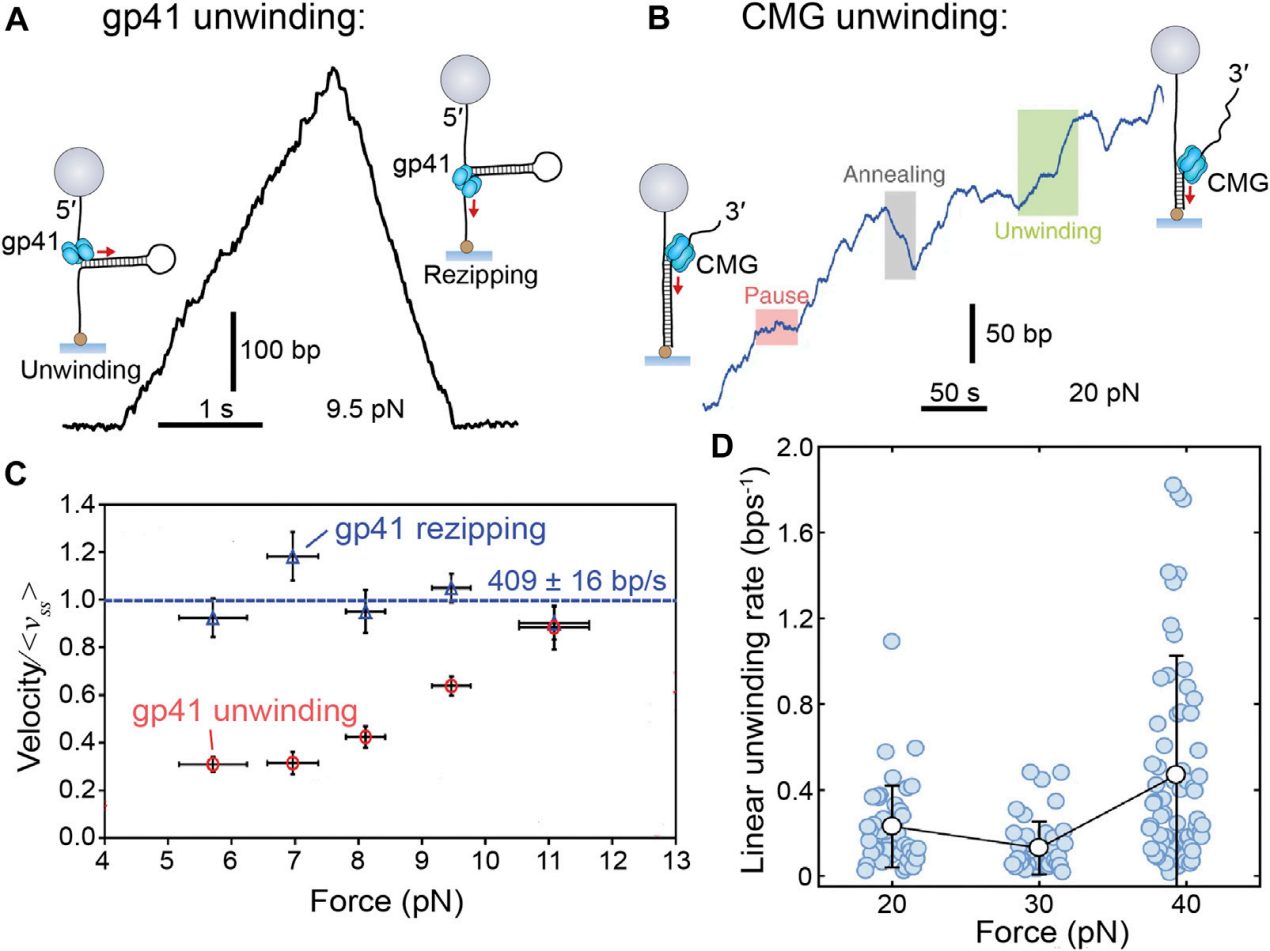
**Single-molecule manipulation of replicative helicase unwinding events. (A)** Magnetic trap measurement of a single T4 gp41 helicase unwinding and rezipping a hairpin DNA template. **(B)** A single CMG unwinding event as measured by a magnetic trap shows more sporadic unwinding, but no rezipping. **(C)** Gp41 unwinding and rezipping rates measured at different forces. Rezipping is independent of force, but equivalent to the ssDNA translocation rate (<*v*_ss_> = 409 ± 16 bp/s) and unwinding rate increases with force (Ribeck and Saleh, 2013). **(D)** CMG unwinding rate also increases with force but with a less obvious trend (Burnham et al., 2019).

Each of the mentioned studies enabled detection of single helicases mediating individual unwinding events (Figures 4A,B). Each of the helicases show a relatively consistent unwinding rate interspersed with pauses, except for CMG, which was comparatively more sporadic (see further discussion of CMG in *Eukaryotic (SF6) Helicase Mechanism*). Unwinding is also occasionally followed by “rezipping” of the DNA template in the wake of the helicase. Rezipping presents as the slow, falling edge of bead movement and has been shown to be equivalent to the helicase translocation rate (Lionnet et al., 2007; Manosas et al., 2010). Interestingly, the measured unwinding rate varied among helicases, but each was consistently slower than the known corresponding replication rate (Table 1). Another commonality was the response to increased force applied to the occluded strand (>20 pN) where the unwinding rate (but not rezipping rate) increased exponentially (Figures 4C,D). Forces in this regime are expected to destabilize dsDNA, so therefore it is likely the free energy cost of unwinding at the DNA fork junction is proportionally reduced. Although seemingly removed from the conditions of the cell, this finding has two important implications for helicase unwinding mechanics that will be discussed below.

**TABLE 1:** **Replicative helicase rate and processivity parameters.** For the replicative helicases gp4, gp41, DnaB, and CMG, from T7 phage, T4 phage, *E. coli*, and *S. cerevisiae*, respectively, the best estimates are given for the parameters of unwinding rate, processivity, replication rate, and predicted mode of action.

Helicase	Unwinding rate (bp/s)	Processivity (bp)	Replication rate (bp/s)	Action
Gp4	30 Johnson et al. (2007)	∼400 Johnson et al. (2007)	∼160 Lee et al. (2006)	very weakly active
Gp41	30 Lionnet et al. (2007)	∼200 Lionnet et al. (2007)	∼400 Werner (1968)	passive
DnaB	100 Ribeck et al. (2010)	∼1,000 Ribeck et al. (2010)	300–1,000 Chandler et al. (1975); Tanner et al. (2008)	weakly active
CMG	0.10–0.47 Burnham et al. (2019)	∼800 Burnham et al. (2019)	10–50 Sekedat et al. (2010); Lewis et al. (2020)	weakly active

Firstly, these single-molecule studies confirmed that replicative helicases are inefficient at low forces, but rates comparable to those seen in replication can be obtained by applying a stronger force. Similarly, other single-molecule studies have shown that addition of the polymerase holoenzyme can improve the helicase unwinding rate and processivity (Manosas et al., 2012a; Manosas et al., 2012b). Therefore, a model starts to form of how polymerization acts to destabilize DNA at the fork to promote rapid helicase translocation and unwinding. Further discussion of the synergy between the helicase and polymerase are explored in *Helicase-Polymerase Coupling Interactions*.

The second important implication of the force-unwinding relationship relates to the thermodynamics of helicase-mediated unwinding. The process of ‘unzipping’ the next base pair is the rate-limiting step in the cycle of unwinding and can occur in two possible modes: either passive or active. For passive unwinding, ATP is only consumed for the helicase to translocate along ssDNA, and the DNA junction is advanced following thermal fraying of the proximal base pairs. For active unwinding, the energy from ATP hydrolysis by the helicase contributes to DNA junction destabilization as well as translocation. A dependence on force applied to assist duplex opening is a trait of the passive helicase unwinding model (Betterton and Julicher, 2003; Betterton and Julicher, 2005). Gp41, Gp4, DnaB, and CMG all exhibit this property—in distinct contrast to that of a known active helicase like RecQ (Manosas et al., 2010). Some of these helicases also demonstrate sensitivity to the AT content in the unwound DNA (Ribeck et al., 2010; Ribeck and Saleh, 2013; Syed et al., 2014; Schlierf et al., 2019), which is another trait of passive helicases. Intriguingly, for gp41, the measured force-rate curve is fit nicely by the passive model; however, for gp4 and DnaB, the results deviate from the model at high forces. Thus, it is tempting to argue that these helicases must somehow contribute to DNA junction destabilization and are thus “weakly active”.

A “weakly active” helicase is an enzymatically interesting concept; however, the available data do not unequivocally support this conclusion and its implications for replicative helicases. We should also question the robustness of the passive unwinding model. The model is extremely sensitive to the value of step size and slippage frequency—parameters that are both very difficult to measure. Notably, for gp4 the data fit well with different parameter sets, which actually predict different unwinding mechanisms (Manosas et al., 2010; Chakrabarti et al., 2019). As a consequence, the topic of passive versus active mechanism remains controversial, with the literature rife with conflicting statements about the true unwinding mechanism based on different methods. It is possible that all these replicative helicases are passive, and thus far we have not been able to identify the proper model. Alternatively, some of these helicases could be partially active, but we do not currently possess techniques with the necessary discriminatory power to confirm this hypothesis. In the future we should look for more comprehensive theoretical modelling (see Chakrabarti et al., 2019) and further experimental studies with higher throughput to verify these predictions.

### Subunit Coordination

Unlike monomeric or dimeric helicases, the replicative helicases are all hexameric rings (O'Donnell and Li, 2018) and therefore require a great deal of coordination among subunits to achieve translocation. Through the power of single-molecule methods like smFRET and trapping assays, we are able to observe individual unwinding events mediated by single helicases in real-time with the resolution approaching individual base pairs. From these experiments it is possible to investigate the coordination of hexameric subunits by quantifying and comparing intra- and inter-molecular dynamics during unwinding.

It is due to the use of single-molecule methods that we have observed helicase slippage. By manipulating gp4 at the single-molecule level using an optical trap, Sun et al. (2011) detected that the helicase mostly maintains a constant unwinding rate but occasionally slips backwards on the DNA. This helicase slippage is a potential indicator of desynchronized subunits. The authors also found that gp4 slippage only occurs in the presence of ATP, and not dTTP, the latter being the preferred nucleotide during replication (Matson and Richardson, 1983). Therefore, it is conceivable that ATP-induced slippage is a backup mechanism to keep the helicase close to the polymerase if the polymerase ever slows or stalls when the dNTP pool is depleted and decoupling between the enzymes occurs.

**FIGURE 5:**
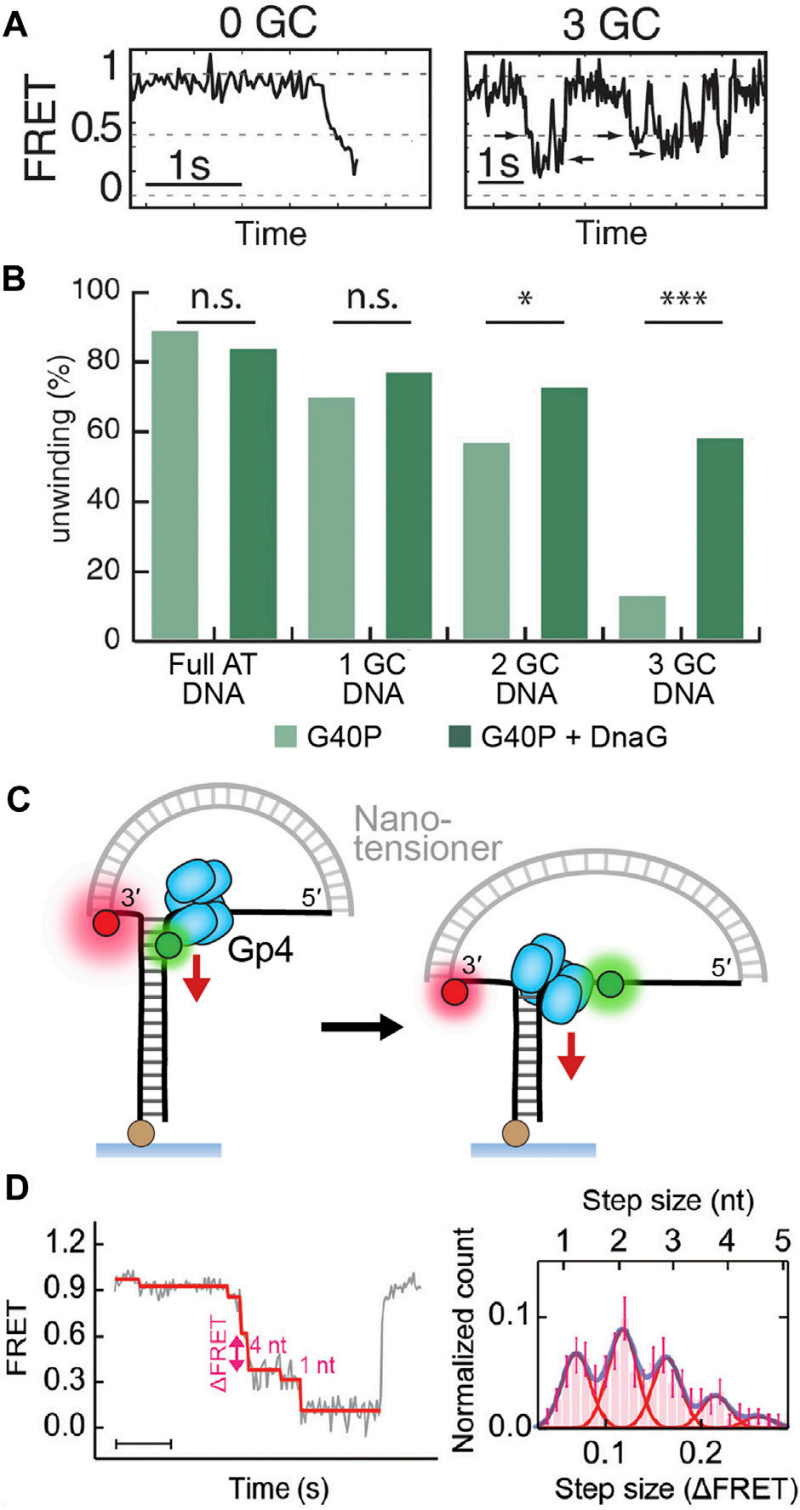
**Detection of replicative helicase stepping using single-molecule FRET. (A)** Typical FRET unwinding traces for G40P, the DnaB-like helicase (Schlierf et al., 2019). On templates containing no GC base pairs, G40P unwinding exhibits a rapid decrease in FRET. On templates containing 3 GC base pairs, G40P frequently stalls (see arrows), slips backwards and then re-attempts unwinding. **(B)** The percentage of FRET templates unwound by G40P decreases with increasing GC DNA content, but a higher percentage can be recovered with the inclusion of the DnaG primase. **(C)** A nanotensioner applied to the single-molecule FRET unwinding assay stabilizes the overhangs of the unwound DNA strands and thus improves the resolution in FRET signal to <1 bp (Lin et al., 2017). **(D)** Typical FRET unwinding traces for the gp4 helicase, where a nanotensioner is incorporated into the DNA template. (Ma et al., 2020). A histogram of gp4 step sizes (right) shows the helicase can sample a hierarchy of steps with 2 nt/step being the most common size.

Slippage is also observed in a single-molecule trapping study of T4 gp41 (Manosas et al., 2012a) and a smFRET study of G40P, the DnaB-like helicase of phage SPP1 (Schlierf et al., 2019). Interestingly, these helicases both demonstrated a higher frequency of slippage at regions of DNA with a high GC content (Figures 5A,B). This aligns with the expectation that replicative helicases passively unzip DNA (as discussed in *Active Versus Passive Helicases*). In this passive model, GC base pairs present a higher free energy barrier for fraying and thus greater chance of failure and subsequent slipping. It is likely that slippage is attributable to either the simultaneous unbinding of all subunits from DNA or a cascading effect after one subunit unbinds. Interestingly, it has been demonstrated that this slippage effect is negated by the presence of the polymerase (Manosas et al., 2012a) or primase (Figure 5B) (Schlierf et al., 2019), suggesting that slippage only ever occurs when the helicase is decoupled from the replisome (see *Replisome Coupling* for further discussion).

There was no detection of CMG helicase slippage when observing unwinding events at the single-molecule level using magnetic traps (Figure 4B) (Burnham et al., 2019). There are several possible explanations for this observation. CMG does interact with several other replisome components, such as Pol ε, Mcm10, and MTC, that could potentially inhibit slippage (reviewed in Lewis and Costa, 2020), but they were not present in this assay. It is possible that the GC content of the DNA template of this assay did not provoke slippage. Another alternative is that the MCM2–7 hetero-hexamer arranges the different subunits around DNA in a way that prevents slipping. However, we do not understand the unwinding mechanism of CMG well enough to pass this assessment (see *Eukaryotic (SF6) Helicase Mechanism*). Alternatively, in structures of CMG, the Cdc45 subunit is in contact with both the MCM2–7 ring and DNA. It has therefore been suggested that this protein acts as an internal brake within the helicase to prevent slippage events (Petojevic et al., 2015). High-resolution single-molecule FRET might be applied in the future to observe CMG activity on DNA templates (Figure 1C; see *Step Size*), to see if the helicase responds differently to variations in the GC content of the template.

### Step Size

During unwinding, helicases separate base pairs in discrete, repeated events known as steps. Thus, it is important to determine the factors that contribute to the stepping action and step size of a helicase. Step size can be further defined as either the “physical” or “kinetic” step size, where the former is the physical distance (in nucleotides) traveled per NTP hydrolyzed, and the latter is the distance between two rate-limiting transitions.

In all structures of the replicative helicases bound to ssDNA (Figure 2), each helicase subunit contacts the backbone of two nucleotides. These structures imply that each of these helicases move with a physical step size of 2 bp per ATP cycle. In contrast, early single-turnover ensemble measurements of the kinetic step size of gp4 resulted in an estimate of ∼10 bp (Jeong et al., 2004). The lab of Taekjip Ha has developed single-molecule assays based on FRET to detect helicase unwinding events with a structural resolution of ∼3 bp (Figure 1C; see *The Single-Molecule Toolbox to Examine Helicases*) (Deniz et al., 1999; Ha et al., 2002). They have since applied this assay to a number of helicases, including gp4 (Syed et al., 2014) and DnaB-like, phage SPP1 G40P (Schlierf et al., 2019). They were able to directly detect small steps of 2–3 bp for both helicases. Interestingly, further analysis of the single-molecule kinetics showed that the dwell time between steps was best described by a gamma distribution rather than an exponential one. Such a fit suggests that there are several rate-limiting kinetic steps hidden within bursts of 2–3 bp of unwinding.

This theory on stepping action was recently proven by direct observation. Lin et al. (2017) were able to further enhance the resolution of the single-molecule FRET unwinding assay with the addition of a nanotensioner. A nanotensioner is a short DNA duplex designed to apply force to both ends of the unwinding template overhangs (Figure 5C). As a result, the fluorophores on the overhangs are stabilized and the FRET resolution is improved to <1 bp (Lin et al., 2017). When used to study gp4, the nanotensioner-enhanced FRET unwinding assay revealed that the helicase took a variety of step sizes between 1–4 bp, with 2 bp being the most common (Figure 4D) (Ma et al., 2020). A possible explanation for the variation in step size is to consider that these hexameric helicases sample a hierarchy of steps where part of the elastic energy from a prior step can contribute to the next step. In their study, Ma et al. also found that gp4 stalled at abasic lesions, where it shuffled 1 bp back and forth and then occasionally jumped past the lesion. The ability to sample different step sizes and ‘leapfrog’ lesions could be a potential mechanism to confer robustness by ensuring the helicase, and by extension the replisome, acts processively to duplicate DNA.

The only replicative helicase not discussed here is CMG. Currently our knowledge of the CMG helicase stepping mechanism is severely limited. Structural data shows the MCM subunits each in contact with 2 nucleotides (Figure 2C), yet the step size could still vary (Georgescu et al., 2017). Furthermore, this and other CMG structures (Abid Ali et al., 2016; Yuan et al., 2016) show CMG arranged in a different quaternary state compared to gp4 and DnaB, so it is challenging to derive any information from the prokaryotic model to predict the likely nucleoprotein stepping dynamics of eukaryotic CMG. Single-molecule assays with high spatial-temporal resolution seem the best path forward to help elucidate the step size of CMG.

### The Mechanisms of Replicative Helicase Translocation and Unwinding

Structural studies have arguably made the greatest contribution to our knowledge of helicase unwinding mechanisms. Atomic-level visualization of helicase structures gives us an idea of the different static architectures of unwinding; however, recent single-molecule studies have been able to report on the intricate dynamics that occur in between these states. The following sub-sections compile our current understanding of unwinding mechanisms and highlight the progress derived from single-molecule studies.

#### Bacterial and Bacteriophage (SF4) Helicase Mechanism

The bacterial and phage replicative helicases are all expected to unwind in the 5′–3′ direction via the same mechanism based on their similarity in structural motifs (as identified in *Phage T4 gp41 and Phage T7 gp4 Helicases and E. coli DnaB Helicase*). The nucleo-protein structures of gp4 (Gao et al., 2019) and DnaB (Itsathitphaisarn et al., 2012) both portray a hexameric spiral staircase of identical subunits around a spiral of ssDNA (Figures 3A,B). Efforts to obtain a structure of gp41 have not been successful, attributable to issues related to protein solubility; however, the architecture is expected to be similar to that of gp4 (Mueser et al., 2010). In the gp4 and DnaB models, the positioning of the DNA-interacting loops and general hexamer flexibility suggests that the subunits might move sequentially in a “hand-over-hand” fashion. There is no robust evidence exactly showing that NTP hydrolysis is reserved for individual subunit movement, or that the process takes place in sequential movements. However, Gao et al. find in their gp4 structures that the NTPase active site is tightest at the last subunit position in the spiral. Therefore, NTP hydrolysis is most likely to occur at this last subunit. We also know from bulk biochemical studies that NTP hydrolysis triggers ssDNA release from gp4 (Hingorani and Patel, 1993). Binding a new NTP then increases ssDNA affinity and permits advancement to the next available site. Therefore, it is not impossible to imagine that these replicative helicases couple NTP hydrolysis to subunit translocation from last to first in a repetitive cycle.

Single-molecule studies have played an important role in increasing our understanding of helicase unwinding. As we identified earlier in this review, single-molecule trapping studies have demonstrated how replicative helicases passively unzip duplex DNA (see *Active Versus Passive Helicases*). Similarly, single-molecule FRET unwinding experiments showed that the prokaryotic helicases most commonly step forward 2 bp at a time but can sample steps of 1–4 bp in size (see *Step Size*). Furthermore, from the analysis of single-molecule unwinding events, we have witnessed helicase slippage occurring as a likely result of transiently desynchronized subunits (see *Subunit Coordination*). Collectively, these studies tell us that the unwinding process has considerable potential for dynamics. Ma et al. (2020) recently examined these dynamic activities in the context of the hand-over-hand mechanism and predict the existence of a possible intermediate state. In their single-molecule work, they find gp4 shuffling back and forth at abasic lesions or at low dTTP concentrations. The available static structures of gp4 cannot explain how this backward movement might be possible. Thus, the authors rationalize that shuffling could happen if the translocation of the last subunit is delayed at low dTTP concentrations, the first subunit also may dissociate so that only four subunits are in contact with DNA, and then the junction can rewind. Further high-resolution structural and dynamic experimentation is needed to support this theory.

#### Eukaryotic (SF6) Helicase Mechanism

Similarly to the bacterial and phage helicases, most of our understanding of eukaryotic replicative helicase mechanism is derived from structural studies. These structures depict CMG as a hetero-hexameric spiral coiled around A-form-like ssDNA with the final two subunits bridging the gap in the spiral (Abid Ali et al., 2016; Georgescu et al., 2017) (Figure 2C). So far CMG has only been found in two distinct conformers, not the six one might expect for a hexamer, leading to proposals that CMG moves in a more complex fashion than its bacterial counterparts. A recent cryo-EM structure identified DNA contacts in the N-tier ring that possibly forms a barrier to exclude the lagging strand during unwinding (Yuan et al., 2020). It is proposed that this “dam-and-diversion” process may explain how steric exclusion occurs for CMG. Unfortunately, our limited knowledge of the unwinding mechanism can be attributed to the relative infancy of the single-molecule investigation of this enzyme.

The one high-resolution single-molecule study of CMG using magnetic traps offers a good starting point and has identified a passive action of unzipping—in line with all the other replicative helicases (Figures 4B,D; see *Active Versus Passive Helicases*) (Burnham et al., 2019). Interestingly, this study also found significant intra-molecular variation in unwinding rate. Other low-resolution single-molecule studies have found CMG unwinding to be enhanced by the presence of replisomal components such as RPA (Kose et al., 2020) and Mcm10 (Wasserman et al., 2019). Interestingly, through the use of correlative fluorescence and force spectroscopy, Wasserman et al. were able to detect instances where CMG transitions between ssDNA translocation to more rapid and random dsDNA diffusion upon applying a duplex destabilizing force. The authors propose a gate must exist in the MCM2–7 hexamer of CMG to permit the move from encircling single-stranded to double-stranded DNA. It is possible such a gate could allow CMG to escape stalled replisomes and restart replication further downstream (Wasserman et al., 2019). This propensity of CMG for variation is unique among replicative helicases and reinforces the idea of a very different mechanism of unwinding.

The best path forward to uncovering more about the CMG unwinding mechanism likely involves further investment in high-resolution single-molecule analyses of this enzyme. CMG has not been observed in a single-molecule FRET unwinding assay and this should be the first step on this path using the existing studies on bacterial and phage helicases as a guide (see *Step Size*) (Syed et al., 2014; Schlierf et al., 2019; Ma et al., 2020). FRET could also be used to study the inter-subunit dynamics of unwinding by exploiting the hetero-hexameric nature of CMG to easily label the appropriate subunits—a method that has already been applied to analyze CMG ring closure at an origin of replication (Ticau et al., 2017).

## Dynamics of Helicase-Replisome Interactions

### Helicase Loading During Replication Initiation

Loading of the replicative helicase is the key step in initiating DNA replication. This process begins with initiator proteins recognizing origins of replication and loading replicative helicases in specific locations, allowing initiation of replication in a bidirectional manner at the correct time. Between species, distinctly different players are involved in initiation, however in each system the process is always tightly controlled. Genetic studies in *E. coli* have shown that if some of the initiation factors are suppressed, unregulated and untimely initiation can occur—often with lethal consequence (Charbon et al., 2018). Ensemble-averaging studies have made tremendous headway to characterize the proteins involved in initiation, but single-molecule techniques hold a greater resolving power capable of identifying the initiation assembly order and potential protein intermediates along the way.

The first example of single-molecule investigation of helicase loading during replication initiation was conducted by Benkovic and coworkers. Their work was able to identify the order of key events during T4 initiation. This process is still ambiguous because it is debated whether the gp41 helicase and gp43 polymerase maintain a physical coupling in replication and thus if their initiation is synchronized (Spacciapoli and Nossal, 1994; Dong et al., 1996; Delagoutte and von Hippel, 2001) (see *Helicase-Polymerase Coupling Interactions*). Previous work has shown that the gp59 helicase loader has strong affinity for ssDNA (Jones et al., 2004). It has been suggested that gp59 along with the gp32 single-stranded DNA-binding protein binds the fork first and then recruits the gp41 helicase. The use of single-molecule colocalization and FRET has demonstrated that gp59-gp32 first binds and locks the gp43 polymerase on ssDNA. Addition of the gp41 helicase results in its loading, which then unlocks the polymerase (Xi et al., 2005a; Xi et al., 2005b; Zhang et al., 2005). Therefore, it seems that the gp59 helicase loader plays a critical role to orchestrate T4 replication initiation and synchronize unwinding and polymerization.

Single-molecule methods have also been useful to untangle the intricate process of replication initiation in eukaryotes (reviewed in detail by Lewis and Costa, 2020). At present, we understand the first stage as “origin licensing”, which is temporally confined to the G1 phase of the cell cycle. First, the origin recognition complex (ORC) binds directly to one of the many origin DNA sequences. Next the accessory initiator proteins Cdc6 and Cdt1 recognize, bind and thus become a “marker” of the helicase loading loci. These two proteins then facilitate the loading of two MCM2–7 rings in a head-to-head, double hexamer orientation. When the cell reaches S phase, the second stage described as “origin firing” begins, in which MCM2–7 is converted into a complete CMG helicase with the addition of the Cdc45 and GINS proteins, and finally processive unwinding is activated by the firing factor, Mcm10.

**FIGURE 6:**
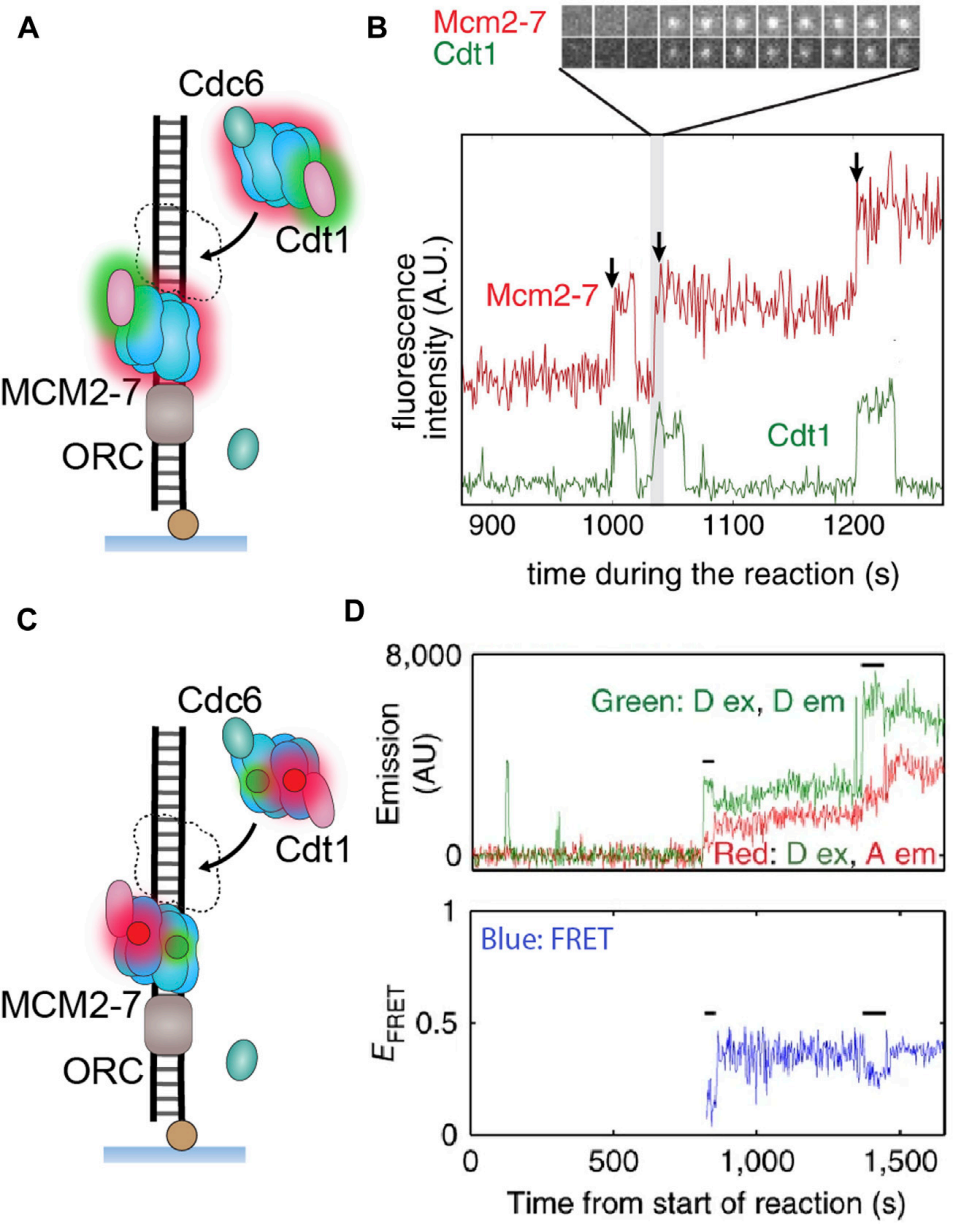
**Single-molecule visualization of CMG assembly during *S. cerevisiae* replication initiation. (A)** A single-molecule assay where two-color colocalization is used to detect CMG loading *in vitro*. **(B)** Fluorescently labeled MCM2–7 (red) binds simultaneously with Cdt1 (green), with multiple hexamers appearing to bind sequentially (Ticau et al., 2015). **(C)** A single-molecule assay where FRET is used to detect MCM2–7 ring opening and closing. **(D)** Observation of both donor and acceptor emission identifies when MCM2–7 binds DNA. The FRET efficiency indicates the hexameric ring state, where high FRET denotes a closed ring and low FRET denotes an open ring. Instances of low FRET (black lines) correlate with MCM2–7 binding, indicating that the ring briefly opens as it encircles DNA during loading (Ticau et al., 2017).

This initiation pathway is highly concerted; however, many features remain ill-defined, with one of the key questions being whether the initial loading of the two helicases occurs simultaneously or sequentially. Ticau et al. answered this question in their single-molecule study when they reconstituted the initiation process *in vitro* and observed loading events using real-time colocalization methods (Figure 1D; see *The Single-Molecule Toolbox to Examine Helicases*) with fluorescently labeled DNA and MCM2–7 hexamers. These assays were able to distinguish sequential fluorescence steps corresponding to MCM2–7 hexamers loading one after the other (Figures 6A,B) (Ticau et al., 2015). Further colocalization experiments showed that each hexamer loading was correlated with the binding of a different set of Cdc6 and Cdt1 proteins (Cdt1 example shown in Figure 6B). These results were able to demonstrate sequential MCM2–7 loading and also suggest the second hexamer loads in a process distinct from the first. Several years later, the same research group built on their original assay by labeling different MCM2–7 subunits with a FRET pair and monitoring ring closure during loading (Ticau et al., 2017). They observed both donor and acceptor fluorescence as an indicator of binding, as well as FRET as a measure of ring opening and closing (Figures 6C,D). The results indicated that as each MCM2–7 hexamer loads, it opens its ring briefly and then closes as it encircles DNA (Figure 5D). This study also identified that ring closure is tightly correlated with ATP hydrolysis and departure of the associated Cdt1 (Ticau et al., 2017).

Other single-molecule studies have been able to corroborate these observations. Specifically, single-molecule use of correlative fluorescence and force spectroscopy made it possible to detect a gate in CMG that opens when the helicase transitions from translocating ssDNA to dsDNA (Wasserman et al., 2019). This study also identified that this gate is distinct from the ring opening mechanism and is possibly how CMG exits the double-hexamer structure during origin firing. Furthermore, Champasa et al. (2019) identified the key residues essential for double-hexamer formation using a combination of mutations and smFRET. In a separate study, Duzdevich et al. (2015) employed a DNA curtain assay to observe hexamer loading within a larger replication initiation pathway. Collectively, these results indicate the mechanism of how replication is controlled to only “fire” once in a bidirectional manner. They also highlight the degree to which replication initiation is a highly coordinated process in eukaryotes.

Interestingly, no single-molecule studies have been done on helicase loading in bacteria. This process has been studied extensively via ensemble biochemical methods and structural analysis (reviewed by Chodavarapu and Kaguni, 2016), yet single-molecule examination could be very useful to clarify the replisome assembly pathway at *oriC* and pinpoint the activation of the DnaB helicase.

### Stability During Replication

All replication complexes need to be highly processive in DNA replication to fully duplicate the long chromosomal molecules. Essentially, this means that the main enzymes of replication, the polymerase and helicase, need to sustain their activity for the entirety of the replication process. Especially for *E. coli*, this expectation of high replisome processivity is based on the known genome size (4.6 Mbp) and its duplication time (∼40 min) (Chandler et al., 1975), coupled with the necessity for only two oppositely-traveling replisomes, but yet the relatively infrequent rate of replication collapse (once in every 5 generations) (Maisnier-Patin et al., 2001). Along with the observation of a relatively stable replication complex, this led to a model of replication where the required high processivity of the replisome was strongly linked to its high stability (Beattie and Reyes-Lamothe, 2015).

**FIGURE 7:**
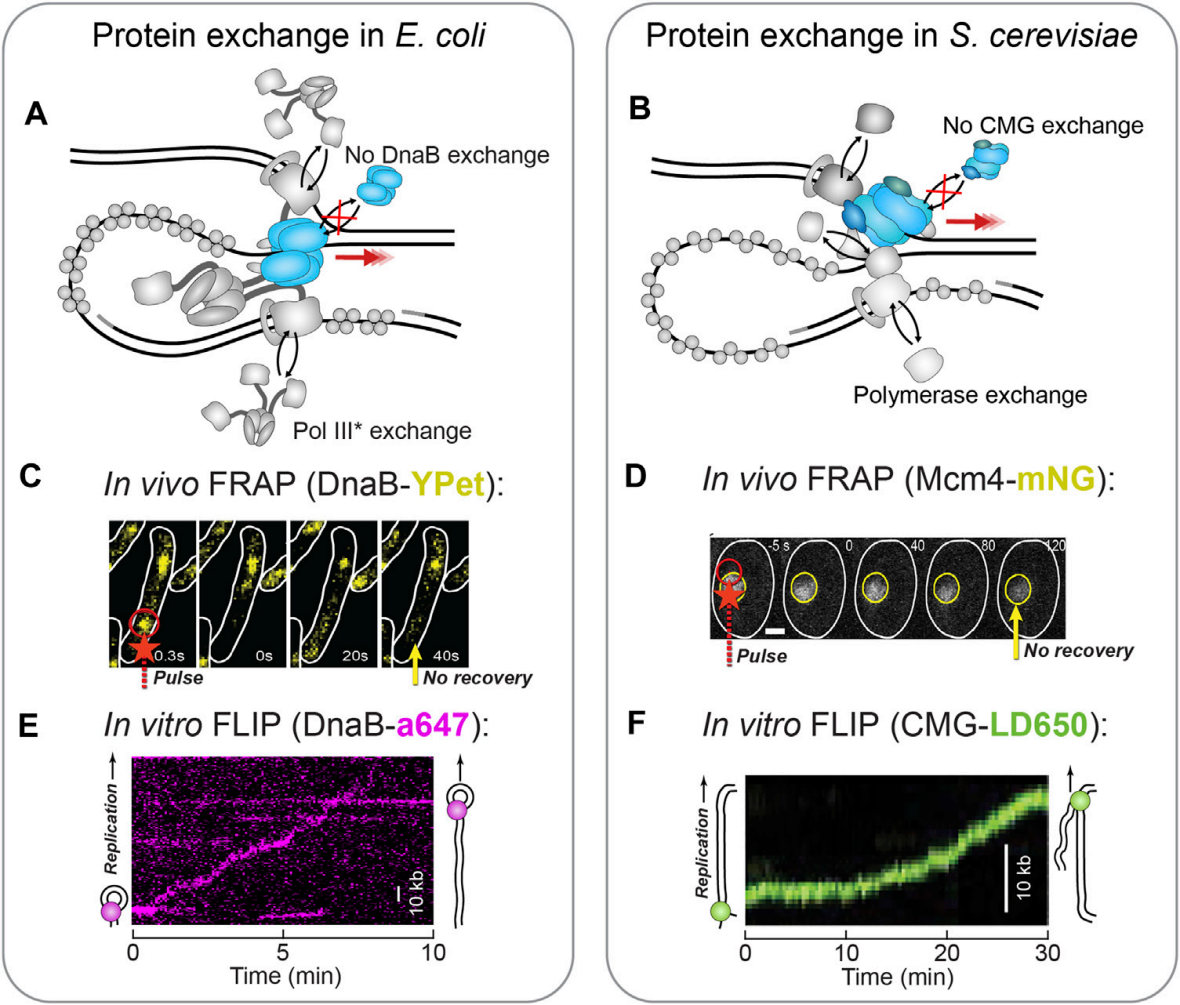
**Single-molecule fluorescence visualization of replisome stability. (A)** In the *E. coli* replisome, the polymerase holoenzyme (polymerase core and clamp loader complex) has been shown to exchange rapidly during replication (Beattie et al., 2017; Lewis et al., 2017), while the DnaB helicase remains stably associated (Spinks et al., 2021). **(B)** In the *S. cerevisiae* replisome, each of the polymerases demonstrates some degree of exchange, while the CMG helicase shows a complete lack of exchange (Kapadia et al., 2020; Lewis et al., 2020). **(C)** *In vivo* FRAP applied to fluorescent DnaB-YPet shows no recovery (yellow arrow) after the FRAP pulse (red arrow) and thus no exchange (Beattie et al., 2017). **(D)** *In vivo* FRAP applied to fluorescent Mcm4-mNG as part of CMG shows no recovery (yellow arrow) in the area bleached by the FRAP pulse (red arrow) (Kapadia et al., 2020). Therefore, CMG does not exchange. **(E)** *In vitro* FLIP applied to fluorescent DnaB-a647 within single-molecule rolling-circle replication. In this example kymograph the DnaB signal persists even though it is challenged with extra unlabeled DnaB, and therefore is not exchanging (Spinks et al., 2021). **(F)** *In vitro* FLIP applied to fluorescent CMG-LD650 during single-molecule replication of a linear DNA template. The CMG signal persists even though it is challenged with excess unlabeled CMG, and thus does not exchange (Lewis et al., 2020).

In contrast to this model however, observations of DNA replication at the single-molecule level revealed that the replisome is not a stable entity but is in fact highly dynamic with components rapidly exchanging in and out. This exchange phenomenon was first hypothesized and detected within the T7 replisome (Loparo et al., 2011; Geertsema et al., 2014), and has since been identified in bacteria (Liao et al., 2016; Beattie et al., 2017; Lewis et al., 2017; Li et al., 2019; Spenkelink et al., 2019; Dubiel et al., 2020) (Figure 7A) and most recently, in eukaryotes (Kapadia et al., 2020; Lewis et al., 2020) (Figure 7B).

In particular, the single-molecule application of FRAP and FLIP have made it possible to measure the kinetics of this exchange process (Figures 1E,F; see *The Single-Molecule Toolbox to Examine Helicases*). In *E. coli*, FRAP applied *in vivo*, at the single-molecule level, to fluorescent versions of the replicative Pol III identified exchange to occur every few seconds (Beattie et al., 2017). Parallel *in vitro* FRAP experiments were able to detect individual *E. coli* Pol III exchange events during *in vitro* rolling-circle DNA replication and found the process to occur in a manner dependent on the concentration of free polymerase (Lewis et al., 2017). A similar effect was identified in *S. cerevisiae*, where again single-molecule FRAP was applied *in vivo* to replisome-bound polymerases (Kapadia et al., 2020) and *in vitro* to polymerases within a replication assay based on linear templates (Lewis et al., 2020); the *in vitro* study demonstrated concentration-dependent polymerase exchange — albeit at a slower rate than observed in *E. coli*.

FLIP is often used to complement FRAP as it measures the loss of fluorescence when a protein exchanges for an unlabeled one. In the cases where FLIP was also used in these studies, it did indeed confirm the results of FRAP (Beattie et al., 2017; Kapadia et al., 2020).

As this dynamic replisome model becomes more solidified in the literature, it raises the question: if the replisome is not stable, how does the complex sustain processive replication? It seems the same single-molecule methods also provide the means to answer this question. Through single-molecule FRAP and FLIP, it has been shown that unlike the polymerases, the replicative helicase exchanges rarely, if at all (Figure 7A). In *E. coli*, *in vivo* FRAP measurements showed fluorescent DnaB never fully recovered its signal, indicative of very stably incorporated helicases (Figure 7C) (Beattie et al., 2017). This observation was corroborated by *in vitro* real-time observations of fluorescent DnaB molecules during rolling-circle replication (Spinks et al., 2021). Here, the DnaB helicase displayed ambiguous recovery in FRAP, but through the use of FLIP, it was shown that once incorporated into the replisome, DnaB is impervious to challenge by a large excess of DnaB molecules in solution (Figure 7E). Comparable observations have been made in *S. cerevisiae*, where fluorescent CMG demonstrated long residence times and low FRAP recovery *in vivo* (Figure 7D) (Kapadia et al., 2020). Also, examination of fluorescent CMG with FLIP during *in vitro* reconstituted replication established that the helicase is unaffected by the presence of excess CMG (Figure 7F) (Lewis et al., 2020). The stability of the phage helicases, gp41 and gp4, have yet to be assessed at the single-molecule level. Yet, it is expected to be consistent with the *E. coli* data owing to their similar replisome architecture.

Collectively, these results suggest that even in different phyla, the replicative helicase very rarely exchanges during DNA replication. Instead, after one helicase is loaded and replication is initiated, it is likely that a single helicase is maintained for the entirety of the replication process. In this sense, the replicative helicase becomes the anchor of the replisome, ensuring processive replication and providing a constant binding site for other exchanging proteins (Figures 7A,B). Furthermore, this high degree of helicase stability is likely the reason for the intricate replication restart pathways that exist across all life to reload the replicative helicase in cases where it has dissociated. It would be interesting to see future work use this kind of single-molecule approach to observe stalled replication forks and examine replisome dynamics in those events.

### Replisome Coupling

In all replisomes, the primary responsibility of the replicative helicase is to unwind double-stranded parental DNA into two template strands for copying. Unlike non-replicative helicases, these replisome-bound helicases also seem to have a secondary role to act as anchor points for the larger multi-protein complex (as discussed in *Stability During Replication*). Interestingly, the interactions between the replicative helicase and other replisomal proteins also appear to enhance helicase activity (as discussed in *Active Versus Passive Helicases*). Observations of replicative helicase activity at the single-molecule level has helped tease out the intricacies of the coupling interactions the replicative helicase has with the replisomal polymerase, primase and single-stranded DNA-binding proteins (SSBs).

#### Helicase-Primase Coupling Interactions

The replisomal primase function is dependent on the replicative helicase, despite these two enzymes apparently moving in opposing directions during primer synthesis. This dependency holds true across all kingdoms, even though the physical form of the primase has diversified. Amongst all this variation, we know relatively little about the underlying principles of the priming process—leaving a substantial space to be explored experimentally, especially with single-molecule methods.

It is from single-molecule studies of helicase-primase interaction that we have been able to detect DNA loops forming during priming for the first time—a process that was previously only thought to occur during Okazaki fragment cycling on the lagging strand (Alberts et al., 1983; Dixon, 2009). It had been speculated that helicase unwinding, and thus the whole replisome, would need to pause or have the primase dissociate during the relatively slow step of primer synthesis, a mechanism that has been observed in a single-molecule hydrodynamic bead assay of T7 replication (Lee et al., 2006). However, later work using the T4 gp41 helicase and gp61 primase within a magnetically trapped bead assay showed that instead of pausing, a loop of lagging-strand DNA (priming loop) is generated during priming by gp61. Only unwinding activity is examined in this assay and unlike the T7 gp4, the T4 helicase and primase are separate entities that can dissociate if need be. Yet if NTPs are present in this assay, the bead moves in a manner characteristic of priming loops (Manosas et al., 2009). Such looping activity is not observed when all priming sites are excluded from the DNA template. Priming loops have also been detected in the T7 system using a combination of ensemble biochemical methods and smFRET (Pandey et al., 2009). More recently a single-molecule hydrodynamic bead assay that enabled the simultaneous detection of T7 phage leading- and lagging-strand synthesis demonstrated that both priming loop formation and pausing during priming can occur during replication (Duderstadt et al., 2016). Altogether, these observations indicate that the replisome employs different mechanisms to coordinate primase synthesis with helicase unwinding, with priming loops the most commonly observed one. Priming loops have not yet been detected in the more complex replisomes of *E. coli* and *S. cerevisiae*, but it would be interesting to know if this mechanism is conserved. Such questions should be answerable using single-molecule approaches.

Another relevant helicase-primase interaction has been uncovered by single-molecule analysis. It appears in some cases that the presence of the primase can prevent backslipping by the helicase (as discussed in *Subunit Coordination*). Such behavior was observed using smFRET of stepping by the DnaB-like G40P helicase. Rapid backslipping of the helicase was shown to occur more frequently at GC junctions and at low ATP concentrations but was fully suppressed with the addition of DnaG primase (Schlierf et al., 2019) (Figure 5B). Interestingly, the measured unwinding rate did not differ with or without DnaG at saturating ATP conditions. These results agree with previous ensemble observations that DnaG has the potential to stimulate helicase activity (Wang et al., 2008; Monachino et al., 2020). Taken together, these studies suggest that the DnaG primase has a stabilizing effect on the helicase which prevents slippage on the DNA.

In a broader context, we are obtaining a clearer picture of how the primase and helicase interact during replication, with each enzyme showing enhanced activity in the presence of the other. It would not be unreasonable to think that these two enzymes have evolved to be co-dependent on each other to act efficiently, but only during DNA replication. In the future it would be interesting to use single-molecule methods to observe the kinetics of primase exchange into and out of the replisome and if dissociation correlates with replication dysfunction. Also, the eukaryotic helicase and primase should be examined. We know from FRAP-based studies that the Pol α-primase exchanges during replication (Lewis et al., 2020), but it is unclear if its main point of contact is the CMG helicase or some other replisome component.

#### Helicase-Polymerase Coupling Interactions

Another important replisomal interaction to consider is that between the helicase and the polymerases. These enzymes are the main driving forces of replication, consuming energy to separate DNA strands and then synthesize new DNA. Bulk biochemical analysis of each enzyme found that both the helicase and polymerase function worse in isolation than within the replisome (summarized in Patel et al., 2011). Based on this observation one can hypothesize that the activities of the helicase and polymerase are tightly coordinated. Single-molecule methods once again are the means to access a deeper level of understanding as to how these enzymes work in combination during replication.

The work of Manosas et al. (2012a) on the coupling of the T4 gp41 helicase and gp43 polymerase is a prime example of the effectiveness of single-molecule tools. They measured enzymatic activity with a magnetically trapped hairpin DNA template and were able to reaffirm the conclusions made from the bulk measurements. Isolated gp41 helicase was measured to unwind slowly (∼100 bp/s for ∼100 bp), and isolated gp43 polymerase is completely inactive as it has no strand displacement activity. Each of these activities improved drastically with the application of force with the polymerase developing a strand-displacement ability at force >10 pN. The external destabilization of the DNA junction leads to ‘replication-like’ rates for both the helicase and polymerase, thus suggesting each enzyme aids the other to progress the fork. The authors confirmed this synergistic effect by combining gp41 and gp43 to carry out leading-strand replication and achieve much faster activity (∼300 bp/s) under normal forces. Interestingly, application of higher force (>9 pN) to replication did not increase activity. Instead, there were clear fast and slow periods within individual trajectories characteristic of rapid helicase unwinding and trailing polymerase synthesis. These observations suggest that during these bursts the helicase and polymerase have become uncoupled from each other. The authors also demonstrated that this uncoupling behavior can also be caused by reducing the dNTP concentration, which likely induces polymerase stalling.

Given that there is no constant physical connection between gp41 and gp43 in the T4 replisome (Delagoutte and von Hippel, 2001; Ishmael et al., 2003), it is probable that the observed coupling and uncoupling is mechanical in nature. Manosas et al. (2012a) identified a unique opportunity to test this theory; they created a chimeric replisome of the T7 polymerase holoenzyme and T4 helicase and inserted it into their single-molecule leading-strand replication assay. They found coupling/uncoupling behavior remarkably similar to the normal T4 replisome, which reinforces their mechanical coupling theory at least for T4.

In the T7 phage system, similar coordinated coupling has been observed in bulk (Notarnicola et al., 1997; Stano et al., 2005; Ghosh et al., 2008; Pandey and Patel, 2014; Nandakumar et al., 2015) and in single-molecule optical trapping studies (Sun et al., 2015, Sun et al., 2018). Interestingly, single-molecule studies also identified that helicase-polymerase synergy promoted movement past DNA lesions and obstructing RNA polymerases (Sun et al., 2015, Sun et al., 2018). In a recent structure, the T7 gp4 helicase and gp5 polymerase are positioned perpendicular to each other at the fork, further suggesting there is some coupling effect during unwinding (Gao et al., 2019); however, mutational evidence suggests that such synergy is not dependent on the physical connection between the helicase and polymerase (Stano et al., 2005). We are yet to see if the T7 replisome is capable of uncoupling—an activity best suited for single-molecule analysis. We are also yet to see if the helicase and polymerase of bacteria and eukaryotes exhibit coupling effects. One single-molecule study of reconstituted *E. coli* rolling-circle replication did identify a potential uncoupling action, with the DnaB helicase continuing unwinding during pauses in replication (Graham et al., 2017). Higher-resolution single-molecule studies are needed to dissect this behavior within *E. coli* DNA replication. Future work should also focus on the mechanistic aspects of helicase-polymerase synergy—specifically to identify triggers of uncoupling and the subsequent factors that determine recoupling.

#### Functional Helicase-SSB Interactions

Single-molecule investigation of replicative helicases has also shown that SSBs have an enhancement effect on unwinding. There is no known direct physical connection between the replicative helicase and SSB within the replisome of any species. Therefore, we can envisage that SSBs assist the helicase either by binding to the translocated strand and preventing backslipping or by sequestering the free excluded strand upon thermal fraying to aid in unwinding. These scenarios are not mutually exclusive, so there is a possibility that both occur during unwinding.

Observations of gp41 helicase unwinding of magnetically trapped hairpin templates identified occasional backslipping, but the addition of the T4 gp32 SSB inhibited all slippage (Manosas et al., 2012a). This study also found gp32 made a moderate improvement (∼50%) in gp41 unwinding rates at low forces. Hence in this case, it seems that gp32 mainly assists gp41 by binding the translocated strand behind the helicase to prevent slipping but can also bind the excluded strand to aid unwinding.

RPA, the eukaryotic replicative SSB was also observed to stimulate CMG helicase unwinding in single-molecule experiments. In this case, the CMG helicase alone unwinds very slowly, but the addition of RPA speeds up the process 10–20-fold (Kose et al., 2020). This rate increase is large compared to the stimulation seen with T4 gp41 unwinding. Interestingly, in the eukaryotic replisome architecture, lagging-strand synthesis occurs on the excluded strand, which consistently positions RPA proximal to CMG at the fork (see replisome; Figure 3D). Thus, it is possible RPA fulfils a larger, auxiliary role in unwinding, similarly to the leading-strand polymerase in prokaryotes (see *Helicase-Polymerase Coupling Interactions*). This theory should be investigated further by high-resolution examination of the rate of replication with and without RPA. Also, a high-resolution structure of CMG unwinding DNA in the presence of RPA would also surely help elucidate the unwinding state within the eukaryotic replisome.

## Conclusion and Outlook

Single-molecule methods are becoming increasingly popular to examine the properties of motor enzymes in general and replicative helicases in particular. Methods such as optical and magnetic traps are becoming the standard to measure the kinetics of these enzymes at the single-molecule level to develop mechanistic information. On the other hand, fluorescence single-molecule techniques provide the means to access replicative helicase dynamics and coupling during each stage of replication both *in vitro* and *in vivo*.

Collectively, the insights of the single-molecule studies discussed in this review indicate that replicative helicases, although considerably diverse, adhere to a set of universal principles. The unwinding mechanism of each of these helicases is an intricate and concerted stepping process; however, they all passively unzip DNA and are poor helicases outside of the replisome. Taken along with the knowledge that each replicative helicase interacts with several other replisome components, this suggests that these enzymes have evolved to be dependent on other replisomal proteins, and vice versa. Also, by means of single-molecule investigation we understand more of the pathways involved in the intricately controlled process of replication initiation. Single-molecule methods have not been applied to *E. coli* initiation, but the potential exists for such research in the future. Likewise, single-molecule analysis of replisomal exchange identified that replicative helicases exchange very infrequently and instead are stably maintained throughout replication. This result suggests that beyond unwinding, replicative helicases also play a role to act as a processivity factor of the replisome, providing a stable platform for other exchanging components.

Looking at the bigger picture, single-molecule methods have added another perspective from which to analyze replicative helicases. Complementing robust ensemble biochemical techniques and structural methods, we have the means to understand these enzymes more comprehensively, and by extension also better understand the replisome.

In the future, we expect to see further advancements in the field of single-molecule replicative helicase research as discussed here. Also, it is likely that emerging single-molecule techniques will further expand the toolbox we have to analyze and understand these helicases. For example, methods such as single-molecule nanopores or hybrid fluorescence and force measurements have been effective to examine other motor enzymes (reviewed in Mohapatra et al., 2020). Single-molecule techniques should also be used to study other more auxiliary aspects of replicative helicases that are relevant at other points of the cell cycle. For example, most of the studies discussed here used DNA without any obstacles. While outside the scope of this review, another interesting area of research is how helicases respond to challenges and roadblocks that exist within the cell. Several recent single-molecule studies have sought to explore this concept (Fu et al., 2011; Yardimci et al., 2012; Mangiameli et al., 2017; Schauer et al., 2020). Many questions remain regarding the different types of challenges faced by replicative helicases operating in a cellular context and single-molecule approaches have the potential to contribute significantly to this important research area.

## References

[B1] Abid Ali F. Renault L. Gannon J. Gahlon H. L. Kotecha A. Zhou J. C. (2016). Cryo-EM Structures of the Eukaryotic Replicative Helicase Bound to a Translocation Substrate. Nat. Commun. 7, 10708. 10.1038/ncomms10708 26888060 PMC4759635

[B2] Alberts B. M. Barry J. Bedinger P. Formosa T. Jongeneel C. V. Kreuzer K. N. (1983). Studies on DNA Replication in the Bacteriophage T4 a--.Gif System. Cold Spring Harbor Symposia Quantitative Biol. 47 (Pt 2), 655–668. 10.1101/sqb.1983.047.01.077 6305581

[B3] Arias-Palomo E. Puri N. O’Shea Murray V. L. Yan Q. Berger J. M. (2019). Physical Basis for the Loading of a Bacterial Replicative Helicase onto DNA. Mol. Cell. 74, 173–184. 10.1016/j.molcel.2019.01.023 30797687 PMC6450724

[B4] Beattie T. R. Kapadia N. Nicolas E. Uphoff S. Wollman A. J. Leake M. C. (2017). Frequent Exchange of the DNA Polymerase during Bacterial Chromosome Replication. eLife 6, 21763. 10.7554/eLife.21763 PMC5403216 28362256

[B5] Beattie T. R. Reyes-Lamothe R. (2015). A Replisome's Journey through the Bacterial Chromosome. Front. Microbiol. 6, 562. 10.3389/fmicb.2015.00562 26097470 PMC4456610

[B6] Benkovic S. J. Spiering M. M. (2017). Understanding DNA Replication by the Bacteriophage T4 Replisome. J. Biol. Chem. 292, 18434–18442. 10.1074/jbc.R117.811208 28972188 PMC5682956

[B7] Betterton M. D. Jülicher F. (2003). A Motor that Makes its Own Track: Helicase Unwinding of DNA. Phys. Rev. Lett. 91, 258103. 10.1103/PhysRevLett.91.258103 14754162

[B8] Betterton M. D. Jülicher F. (2005). Opening of Nucleic-Acid Double Strands by Helicases: Active versus Passive Opening. Phys. Rev. E. 71, 011904. 10.1103/PhysRevE.71.011904 15697627

[B9] Brosh R. M. Jr. Matson S. W. (2020). History of DNA Helicases. Genes 11, 255. 10.3390/genes11030255 PMC7140857 32120966

[B10] Burnham D. R. Kose H. B. Hoyle R. B. Yardimci H. (2019). The Mechanism of DNA Unwinding by the Eukaryotic Replicative Helicase. Nat. Commun. 10, 2159. 10.1038/s41467-019-09896-2 31089141 PMC6517413

[B11] Chakrabarti S. Jarzynski C. Thirumalai D. (2019). Processivity, Velocity, and Universal Characteristics of Nucleic Acid Unwinding by Helicases. Biophysical J. 117, 867–879. 10.1016/j.bpj.2019.07.021 PMC6731385 31400912

[B12] Champasa K. Blank C. Friedman L. J. Gelles J. Bell S. P. (2019). A Conserved Mcm4 Motif Is Required for Mcm2-7 Double-Hexamer Formation and Origin DNA Unwinding. eLife 8, 45538. 10.7554/eLife.45538 PMC6701924 31385807

[B13] Chandler M. Bird R. E. Caro L. (1975). The Replication Time of the *Escherichia coli* K12 Chromosome as a Function of Cell Doubling Time. J. Mol. Biol. 94, 127–132. 10.1016/0022-2836(75)90410-6 1095767

[B14] Charbon G. Riber L. Løbner-Olesen A. (2018). Countermeasures to Survive Excessive Chromosome Replication in *Escherichia coli* . Curr. Genet. 64, 71–79. 10.1007/s00294-017-0725-4 28664289

[B15] Chodavarapu S. Kaguni J. M. (2016). Replication Initiation in Bacteria. Enzymes 39, 1–30. 10.1016/bs.enz.2016.03.001 27241926 PMC5551690

[B16] Delagoutte E. von Hippel P. H. (2002). Helicase Mechanisms and the Coupling of Helicases within Macromolecular Machines Part I: Structures and Properties of Isolated Helicases. Quart. Rev. Biophys. 35, 431–478. 10.1017/s0033583502003852 12621862

[B17] Delagoutte E. von Hippel P. H. (2003). Helicase Mechanisms and the Coupling of Helicases within Macromolecular Machines Part II: Integration of Helicases into Cellular Processes. Quart. Rev. Biophys. 36, 1–69. 10.1017/s0033583502003864 12643042

[B18] Delagoutte E. von Hippel P. H. (2001). Molecular Mechanisms of the Functional Coupling of the Helicase (Gp41) and Polymerase (Gp43) of Bacteriophage T4 within the DNA Replication Fork. Biochemistry 40, 4459–4477. 10.1021/bi001306l 11284703

[B19] Deniz A. A. Dahan M. Grunwell J. R. Ha T. Faulhaber A. E. Chemla D. S. (1999). Single-pair Fluorescence Resonance Energy Transfer on Freely Diffusing Molecules: Observation of Forster Distance Dependence and Subpopulations. Proc. Natl. Acad. Sci. 96, 3670–3675. 10.1073/pnas.96.7.3670 10097095 PMC22352

[B20] Dixon N. E. (2009). Prime-time Looping. Nature 462, 854–855. 10.1038/462854a 20016583

[B21] Dong F. Weitzel S. E. von Hippel P. H. (1996). A Coupled Complex of T4 DNA Replication Helicase (Gp41) and Polymerase (Gp43) Can Perform Rapid and Processive DNA Strand-Displacement Synthesis. Proc. Natl. Acad. Sci. 93, 14456–14461. 10.1073/pnas.93.25.14456 8962073 PMC26154

[B22] Dubiel K. Henry C. Spenkelink L. M. Kozlov A. G. Wood E. A. Jergic S. (2020). Development of a Single-Stranded DNA-Binding Protein Fluorescent Fusion Toolbox. Nucleic Acids Res. 48, 6053–6067. 10.1093/nar/gkaa320 32374866 PMC7293020

[B23] Duderstadt K. E. Geertsema H. J. Stratmann S. A. Punter C. M. Kulczyk A. W. Richardson C. C. (2016). Simultaneous Real-Time Imaging of Leading and Lagging Strand Synthesis Reveals the Coordination Dynamics of Single Replisomes. Mol. Cell. 64, 1035–1047. 10.1016/j.molcel.2016.10.028 27889453

[B24] Duzdevich D. Warner M. D. Ticau S. Ivica N. A. Bell S. P. Greene E. C. (2015). The Dynamics of Eukaryotic Replication Initiation: Origin Specificity, Licensing, and Firing at the Single-Molecule Level. Mol. Cell. 58, 483–494. 10.1016/j.molcel.2015.03.017 25921072 PMC4427541

[B25] Fu Y. V. Yardimci H. Long D. T. Guainazzi A. Bermudez V. P. Hurwitz J. (2011). Selective Bypass of a Lagging Strand Roadblock by the Eukaryotic Replicative DNA Helicase. Cell 146, 931–941. 10.1016/j.cell.2011.07.045 21925316 PMC3209622

[B26] Gao Y. Cui Y. Fox T. Lin S. Wang H. de Val N. (2019). Structures and Operating Principles of the Replisome. Science 363, eaav7003. 10.1126/science.aav7003 30679383 PMC6681829

[B27] Geertsema H. J. Kulczyk A. W. Richardson C. C. van Oijen A. M. (2014). Single-molecule Studies of Polymerase Dynamics and Stoichiometry at the Bacteriophage T7 Replication Machinery. Proc. Natl. Acad. Sci. 111, 4073–4078. 10.1073/pnas.1402010111 24591606 PMC3964090

[B28] Georgescu R. Yuan Z. Bai L. de Luna Almeida Santos R. Sun J. Zhang D. (2017). Structure of Eukaryotic CMG Helicase at a Replication fork and Implications to Replisome Architecture and Origin Initiation. Proc. Natl. Acad. Sci. U.S.A. 114, E697–E706. 10.1073/pnas.1620500114 28096349 PMC5293012

[B29] Ghosh S. Hamdan S. M. Cook T. E. Richardson C. C. (2008). Interactions of *Escherichia coli* Thioredoxin, the Processivity Factor, with Bacteriophage T7 DNA Polymerase and Helicase. J. Biol. Chem. 283, 32077–32084. 10.1074/jbc.M805062200 18757858 PMC2581581

[B30] Graham J. E. Marians K. J. Kowalczykowski S. C. (2017). Independent and Stochastic Action of DNA Polymerases in the Replisome. Cell 169, 1201–1213. 10.1016/j.cell.2017.05.041 28622507 PMC5548433

[B31] Ha T. Rasnik I. Cheng W. Babcock H. P. Gauss G. H. Lohman T. M. (2002). Initiation and Re-initiation of DNA Unwinding by the *Escherichia coli* Rep Helicase. Nature 419, 638–641. 10.1038/nature01083 12374984

[B32] Hingorani M. M. Patel S. S. (1993). Interactions of Bacteriophage T7 DNA Primase/helicase Protein with Single-Stranded and Double-Stranded DNAs. Biochemistry 32, 12478–12487. 10.1021/bi00097a028 8241139

[B33] Ishmael F. T. Trakselis M. A. Benkovic S. J. (2003). Protein-Protein Interactions in the Bacteriophage T4 Replisome. J. Biol. Chem. 278, 3145–3152. 10.1074/jbc.M209858200 12427736

[B34] Itsathitphaisarn O. Wing R. A. Eliason W. K. Wang J. Steitz T. A. (2012). The Hexameric Helicase DnaB Adopts a Nonplanar Conformation during Translocation. Cell 151, 267–277. 10.1016/j.cell.2012.09.014 23022319 PMC3597440

[B35] Jeong Y.-J. Levin M. K. Patel S. S. (2004). The DNA-Unwinding Mechanism of the Ring Helicase of Bacteriophage T7. Proc. Natl. Acad. Sci. 101, 7264–7269. 10.1073/pnas.0400372101 15123793 PMC409907

[B36] Johnson D. S. Bai L. Smith B. Y. Patel S. S. Wang M. D. (2007). Single-molecule Studies Reveal Dynamics of DNA Unwinding by the Ring-Shaped T7 Helicase. Cell 129, 1299–1309. 10.1016/j.cell.2007.04.038 17604719 PMC2699903

[B37] Jones C. E. Green E. M. Stephens J. A. Mueser T. C. Nossal N. G. (2004). Mutations of Bacteriophage T4 59 Helicase Loader Defective in Binding fork DNA and in Interactions with T4 32 Single-Stranded DNA-Binding Protein. J. Biol. Chem. 279, 25721–25728. 10.1074/jbc.M402128200 15084598

[B38] Kapadia N. El-Hajj Z. W. Zheng H. Beattie T. R. Yu A. Reyes-Lamothe R. (2020). Processive Activity of Replicative DNA Polymerases in the Replisome of Live Eukaryotic Cells. Mol. Cell. 80, 114–126. 10.1016/j.molcel.2020.08.014 32916094

[B39] Kose H. B. Xie S. Cameron G. Strycharska M. S. Yardimci H. (2020). Duplex DNA Engagement and RPA Oppositely Regulate the DNA-Unwinding Rate of CMG Helicase. Nat. Commun. 11, 3713. 10.1038/s41467-020-17443-7 32709841 PMC7382467

[B40] Kulczyk A. W. Richardson C. C. (2016). The Replication System of Bacteriophage T7. Enzymes 39, 89–136. 10.1016/bs.enz.2016.02.001 27241928

[B41] Lee J.-B. Hite R. K. Hamdan S. M. Sunney Xie X. Richardson C. C. van Oijen A. M. (2006). DNA Primase Acts as a Molecular Brake in DNA Replication. Nature 439, 621–624. 10.1038/nature04317 16452983

[B42] Leipe D. D. Aravind L. Koonin E. V. (1999). Did DNA Replication Evolve Twice Independently? Nucleic Acids Res. 27, 3389–3401. 10.1093/nar/27.17.3389 10446225 PMC148579

[B43] Lewis J. S. Costa A. (2020). Caught in the Act: Structural Dynamics of Replication Origin Activation and fork Progression. Biochem. Soc. Trans. 48, 1057–1066. 10.1042/BST20190998 32369549 PMC7329347

[B44] Lewis J. S. Jergic S. Dixon N. E. (2016). The *E. coli* DNA Replication fork. Enzymes 39, 31–88. 10.1016/bs.enz.2016.04.001 27241927

[B45] Lewis J. S. Spenkelink L. M. Jergic S. Wood E. A. Monachino E. Horan N. P. (2017). Single-molecule Visualization of Fast Polymerase Turnover in the Bacterial Replisome. eLife 6, 23932. 10.7554/eLife.23932 PMC5419744 28432790

[B46] Lewis J. S. Spenkelink L. M. Schauer G. D. Yurieva O. Mueller S. H. Natarajan V. (2020). Tunability of DNA Polymerase Stability during Eukaryotic DNA Replication. Mol. Cell. 77, 17–25. 10.1016/j.molcel.2019.10.005 31704183 PMC6943181

[B47] Li Y. Chen Z. Matthews L. A. Simmons L. A. Biteen J. S. (2019). Dynamic Exchange of Two Essential DNA Polymerases during Replication and after fork Arrest. Biophysical J. 116, 684–693. 10.1016/j.bpj.2019.01.008 PMC6382952 30686488

[B48] Liao Y. Li Y. Schroeder J. W. Simmons L. A. Biteen J. S. (2016). Single-molecule DNA Polymerase Dynamics at a Bacterial Replisome in Live Cells. Biophysical J. 111, 2562–2569. 10.1016/j.bpj.2016.11.006 PMC5192695 28002733

[B49] Lin W. Ma J. Nong D. Xu C. Zhang B. Li J. (2017). Helicase Stepping Investigated with One-Nucleotide Resolution Fluorescence Resonance Energy Transfer. Phys. Rev. Lett. 119, 138102. 10.1103/PhysRevLett.119.138102 29341672

[B50] Lionnet T. Spiering M. M. Benkovic S. J. Bensimon D. Croquette V. (2007). Real-time Observation of Bacteriophage T4 Gp41 Helicase Reveals an Unwinding Mechanism. Proc. Natl. Acad. Sci. 104, 19790–19795. 10.1073/pnas.0709793104 18077411 PMC2148377

[B51] Loparo J. J. Kulczyk A. W. Richardson C. C. van Oijen A. M. (2011). Simultaneous Single-Molecule Measurements of Phage T7 Replisome Composition and Function Reveal the Mechanism of Polymerase Exchange. Proc. Natl. Acad. Sci. U.S.A. 108, 3584–3589. 10.1073/pnas.1018824108 21245349 PMC3048139

[B52] Ma J.-B. Chen Z. Xu C.-H. Huang X.-Y. Jia Q. Zou Z.-Y. (2020). Dynamic Structural Insights into the Molecular Mechanism of DNA Unwinding by the Bacteriophage T7 Helicase. Nucleic Acids Res. 48, 3156–3164. 10.1093/nar/gkaa057 32009150 PMC7102974

[B53] Maisnier-Patin S. Nordström K. Dasgupta S. (2001). Replication Arrests during a Single Round of Replication of the *Escherichia coli* Chromosome in the Absence of DnaC Activity. Mol. Microbiol. 42, 1371–1382. 10.1046/j.1365-2958.2001.02718.x 11886566

[B54] Mangiameli S. M. Merrikh C. N. Wiggins P. A. Merrikh H. (2017). Transcription Leads to Pervasive Replisome Instability in Bacteria. eLife 6, 19848. 10.7554/eLife.19848 PMC5305214 28092263

[B55] Manosas M. Spiering M. M. Ding F. Bensimon D. Allemand J.-F. Benkovic S. J. (2012b). Mechanism of Strand Displacement Synthesis by DNA Replicative Polymerases. Nucleic Acids Res. 40, 6174–6186. 10.1093/nar/gks253 22434889 PMC3401438

[B56] Manosas M. Spiering M. M. Ding F. Croquette V. Benkovic S. J. (2012a). Collaborative Coupling between Polymerase and Helicase for Leading-Strand Synthesis. Nucleic Acids Res. 40, 6187–6198. 10.1093/nar/gks254 22434886 PMC3401439

[B57] Manosas M. Spiering M. M. Zhuang Z. Benkovic S. J. Croquette V. (2009). Coupling DNA Unwinding Activity with Primer Synthesis in the Bacteriophage T4 Primosome. Nat. Chem. Biol. 5, 904–912. 10.1038/nchembio.236 19838204 PMC2784132

[B58] Manosas M. Xi X. G. Bensimon D. Croquette V. (2010). Active and Passive Mechanisms of Helicases. Nucleic Acids Res. 38, 5518–5526. 10.1093/nar/gkq273 20423906 PMC2938219

[B59] Matson S. W. Richardson C. C. (1983). DNA-dependent Nucleoside 5'-triphosphatase Activity of the Gene 4 Protein of Bacteriophage T7. J. Biol. Chem. 258, 14009–14016. 10.1016/s0021-9258(17)44017-8 6139375

[B60] Miller H. Zhou Z. Shepherd J. Wollman A. J. M. Leake M. C. (2018). Single-molecule Techniques in Biophysics: a Review of the Progress in Methods and Applications. Rep. Prog. Phys. 81, 024601. 10.1088/1361-6633/aa8a02 28869217

[B61] Mohapatra S. Lin C.-T. Feng X. A. Basu A. Ha T. (2020). Single-molecule Analysis and Engineering of DNA Motors. Chem. Rev. 120, 36–78. 10.1021/acs.chemrev.9b00361 31661246

[B62] Monachino E. Jergic S. Lewis J. S. Xu Z.-Q. Lo A. T. Y. O’Shea V. L. (2020). A Primase-Induced Conformational Switch Controls the Stability of the Bacterial Replisome. Mol. Cell. 79, 140–154. 10.1016/j.molcel.2020.04.037 32464091 PMC7335327

[B63] Mueser T. C. Hinerman J. M. Devos J. M. Boyer R. A. Williams K. J. (2010). Structural Analysis of Bacteriophage T4 DNA Replication: a Review in the Virology Journal Series on Bacteriophage T4 and its Relatives. Virol. J. 7, 359. 10.1186/1743-422X-7-359 21129204 PMC3012046

[B64] Nandakumar D. Pandey M. Patel S. S. (2015). Cooperative Base Pair Melting by Helicase and Polymerase Positioned One Nucleotide from Each Other. eLife 4, 06562. 10.7554/eLife.06562 PMC4460406 25970034

[B65] Notarnicola S. M. Mulcahy H. L. Lee J. Richardson C. C. (1997). The Acidic Carboxyl Terminus of the Bacteriophage T7 Gene 4 Helicase/primase Interacts with T7 DNA Polymerase. J. Biol. Chem. 272, 18425–18433. 10.1074/jbc.272.29.18425 9218486

[B66] O’Donnell M. E. Li H. (2018). The Ring-Shaped Hexameric Helicases that Function at DNA Replication forks. Nat. Struct. Mol. Biol. 25, 122–130. 10.1038/s41594-018-0024-x 29379175 PMC5876725

[B67] Pandey M. Patel S. S. (2014). Helicase and Polymerase Move Together Close to the fork junction and Copy DNA in One-Nucleotide Steps. Cell Rep. 6, 1129–1138. 10.1016/j.celrep.2014.02.025 24630996 PMC4010093

[B68] Pandey M. Syed S. Donmez I. Patel G. Ha T. Patel S. S. (2009). Coordinating DNA Replication by Means of Priming Loop and Differential Synthesis Rate. Nature 462, 940–943. 10.1038/nature08611 19924126 PMC2896039

[B69] Patel S. S. Pandey M. Nandakumar D. (2011). Dynamic Coupling between the Motors of DNA Replication: Hexameric Helicase, DNA Polymerase, and Primase. Curr. Opin. Chem. Biol. 15, 595–605. 10.1016/j.cbpa.2011.08.003 21865075 PMC3189298

[B70] Perera H. M. Behrmann M. S. Hoang J. M. Griffin W. C. Trakselis M. A. (2019). Contacts and Context that Regulate DNA Helicase Unwinding and Replisome Progression. Enzymes 45, 183–223. 10.1016/bs.enz.2019.08.001 31627877

[B71] Petojevic T. Pesavento J. J. Costa A. Liang J. Wang Z. Berger J. M. (2015). Cdc45 (Cell Division Cycle Protein 45) Guards the Gate of the Eukaryote Replisome Helicase Stabilizing Leading Strand Engagement. Proc. Natl. Acad. Sci. U.S.A. 112, E249–E258. 10.1073/pnas.1422003112 25561522 PMC4311868

[B72] Ribeck N. Kaplan D. L. Bruck I. Saleh O. A. (2010). DnaB Helicase Activity Is Modulated by DNA Geometry and Force. Biophysical J. 99, 2170–2179. 10.1016/j.bpj.2010.07.039 PMC3042577 20923651

[B73] Ribeck N. Saleh O. A. (2013). DNA Unwinding by Ring-Shaped T4 Helicase Gp41 Is Hindered by Tension on the Occluded Strand. PLoS One 8, e79237. 10.1371/journal.pone.0079237 24250825 PMC3826741

[B74] Schauer G. D. Spenkelink L. M. Lewis J. S. Yurieva O. Mueller S. H. van Oijen A. M. (2020). Replisome Bypass of a Protein-Based R-Loop Block by Pif1. Proc. Natl. Acad. Sci. U.S.A. 117, 30354–30361. 10.1073/pnas.2020189117 33199603 PMC7720201

[B75] Scherr M. J. Safaric B. Duderstadt K. E. (2018). Noise in the Machine: Alternative Pathway Sampling Is the Rule during DNA Replication. Bioessays 40, 1700159. 10.1002/bies.201700159 29282758

[B76] Schlierf M. Wang G. Chen X. S. Ha T. (2019). Hexameric Helicase G40P Unwinds DNA in Single Base Pair Steps. eLife 8, e42001. 10.7554/eLife.42001 30688211 PMC6370340

[B77] Sekedat M. D. Fenyö D. Rogers R. S. Tackett A. J. Aitchison J. D. Chait B. T. (2010). GINS Motion Reveals Replication fork Progression Is Remarkably Uniform throughout the Yeast Genome. Mol. Syst. Biol. 6, 353. 10.1038/msb.2010.8 20212525 PMC2858444

[B78] Spacciapoli P. Nossal N. G. (1994). Interaction of DNA Polymerase and DNA Helicase within the Bacteriophage T4 DNA Replication Complex. Leading Strand Synthesis by the T4 DNA Polymerase Mutant A737V (*ts*L141) Requires the T4 Gene 59 Helicase Assembly Protein. J. Biol. Chem. 269, 447–455. 10.1016/s0021-9258(17)42371-4 8276834

[B79] Spenkelink L. M. Lewis J. S. Jergic S. Xu Z.-Q. Robinson A. Dixon N. E. (2019). Recycling of Single-Stranded DNA-Binding Protein by the Bacterial Replisome. Nucleic Acids Res. 47, 4111–4123. 10.1093/nar/gkz090 30767010 PMC6486552

[B80] Spinks R. R. Spenkelink L. M. Stratmann S. A. Xu Z.-Q. Stamford N. P. J. Brown S. E. (2021). DnaB Helicase Dynamics in Bacterial DNA Replication Resolved by Single-Molecule Studies. Nucleic Acids Res. 49, 6804–6816. 10.1093/nar/gkab493 34139009 PMC8266626

[B81] Stano N. M. Jeong Y.-J. Donmez I. Tummalapalli P. Levin M. K. Patel S. S. (2005). DNA Synthesis Provides the Driving Force to Accelerate DNA Unwinding by a Helicase. Nature 435, 370–373. 10.1038/nature03615 15902262 PMC1563444

[B82] Sun B. Johnson D. S. Patel G. Smith B. Y. Pandey M. Patel S. S. (2011). ATP-induced Helicase Slippage Reveals Highly Coordinated Subunits. Nature 478, 132–135. 10.1038/nature10409 21927003 PMC3190587

[B83] Sun B. Pandey M. Inman J. T. Yang Y. Kashlev M. Patel S. S. (2015). T7 Replisome Directly Overcomes DNA Damage. Nat. Commun. 6, 10260. 10.1038/ncomms10260 26675048 PMC4703881

[B84] Sun B. Singh A. Sultana S. Inman J. T. Patel S. S. Wang M. D. (2018). Helicase Promotes Replication Re-initiation from an RNA Transcript. Nat. Commun. 9, 2306. 10.1038/s41467-018-04702-x 29899338 PMC5997990

[B85] Syed S. Pandey M. Patel S. S. Ha T. (2014). Single-molecule Fluorescence Reveals the Unwinding Stepping Mechanism of Replicative Helicase. Cell Rep. 6, 1037–1045. 10.1016/j.celrep.2014.02.022 24630993 PMC3988844

[B86] Tanner N. A. Hamdan S. M. Jergic S. Loscha K. V. Schaeffer P. M. Dixon N. E. (2008). Single-molecule Studies of fork Dynamics in *Escherichia coli* DNA Replication. Nat. Struct. Mol. Biol. 15, 170–176. 10.1038/nsmb.1381 18223657 PMC2651573

[B87] Ticau S. Friedman L. J. Champasa K. Corrêa I. R. Jr. Gelles J. Bell S. P. (2017). Mechanism and Timing of Mcm2-7 Ring Closure during DNA Replication Origin Licensing. Nat. Struct. Mol. Biol. 24, 309–315. 10.1038/nsmb.3375 28191892 PMC5336523

[B88] Ticau S. Friedman L. J. Ivica N. A. Gelles J. Bell S. P. (2015). Single-molecule Studies of Origin Licensing Reveal Mechanisms Ensuring Bidirectional Helicase Loading. Cell 161, 513–525. 10.1016/j.cell.2015.03.012 25892223 PMC4445235

[B89] van Oijen A. M. Dixon N. E. (2015). Probing Molecular Choreography through Single-Molecule Biochemistry. Nat. Struct. Mol. Biol. 22, 948–952. 10.1038/nsmb.3119 26643847

[B90] Wang G. Klein M. G. Tokonzaba E. Zhang Y. Holden L. G. Chen X. S. (2008). The Structure of a DnaB-Family Replicative Helicase and its Interactions with Primase. Nat. Struct. Mol. Biol. 15, 94–100. 10.1038/nsmb1356 18157148

[B91] Wasserman M. R. Schauer G. D. O’Donnell M. E. Liu S. (2019). Replication fork Activation Is Enabled by a Single-Stranded DNA Gate in CMG Helicase. Cell 178, 600–611. 10.1016/j.cell.2019.06.032 31348887 PMC6705614

[B92] Werner R. (1968). Distribution of Growing Points in DNa of Bacteriophage T4. J. Mol. Biol. 33, 679–692. 10.1016/0022-2836(68)90313-6 4882615

[B93] Xi J. Zhang Z. Zhuang Z. Yang J. Spiering M. M. Hammes G. G. (2005a). Interaction between the T4 Helicase Loading Protein (Gp59) and the DNA Polymerase (Gp43): Unlocking of the gp59−gp43−DNA Complex to Initiate Assembly of A Fully Functional Replisome. Biochemistry 44, 7747–7756. 10.1021/bi047296w 15909989

[B94] Xi J. Zhuang Z. Zhang Z. Selzer T. Spiering M. M. Hammes G. G. (2005b). Interaction between the T4 Helicase-Loading Protein (Gp59) and the DNA Polymerase (Gp43): A Locking Mechanism to Delay Replication during Replisome Assembly. Biochemistry 44, 2305–2318. 10.1021/bi0479508 15709743

[B95] Yao N. O'Donnell M. (2016b). Bacterial and Eukaryotic Replisome Machines. JSM Biochem. Mol. Biol. 3 (1), 1013. PMID: 28042596 28042596 PMC5199024

[B96] Yao N. Y. O’Donnell M. E. (2016a). Evolution of Replication Machines. Crit. Rev. Biochem. Mol. Biol. 51, 135–149. 10.3109/10409238.2015.1125845 27160337 PMC4979536

[B97] Yardimci H. Wang X. Loveland A. B. Tappin I. Rudner D. Z. Hurwitz J. (2012). Bypass of a Protein Barrier by a Replicative DNA Helicase. Nature 492, 205–209. 10.1038/nature11730 23201686 PMC3521859

[B98] Yuan Z. Bai L. Sun J. Georgescu R. Liu J. O'Donnell M. E. (2016). Structure of the Eukaryotic Replicative CMG Helicase Suggests a Pumpjack Motion for Translocation. Nat. Struct. Mol. Biol. 23, 217–224. 10.1038/nsmb.3170 26854665 PMC4812828

[B99] Yuan Z. Georgescu R. Bai L. Zhang D. Li H. O’Donnell M. E. (2020). DNA Unwinding Mechanism of a Eukaryotic Replicative CMG Helicase. Nat. Commun. 11, 688. 10.1038/s41467-020-14577-6 32019936 PMC7000775

[B100] Zhang Z. Spiering M. M. Trakselis M. A. Ishmael F. T. Xi J. Benkovic S. J. (2005). Assembly of the Bacteriophage T4 Primosome: Single-Molecule and Ensemble Studies. Proc. Natl. Acad. Sci. 102, 3254–3259. 10.1073/pnas.0500327102 15728347 PMC552937

